# Excited State Structural Evolution of a GFP Single-Site Mutant Tracked by Tunable Femtosecond-Stimulated Raman Spectroscopy

**DOI:** 10.3390/molecules23092226

**Published:** 2018-09-01

**Authors:** Longteng Tang, Liangdong Zhu, Miles A. Taylor, Yanli Wang, S. James Remington, Chong Fang

**Affiliations:** 1 Department of Chemistry, Oregon State University, Corvallis, OR 97331, USA; tanglo@oregonstate.edu (L.T.); zhul@oregonstate.edu (L.Z.); taylormi@oregonstate.edu (M.A.T.); wangyanl@oregonstate.edu (Y.W.); 2 Institute of Molecular Biology and Department of Physics, University of Oregon, Eugene, OR 97403, USA; jreming@uoregon.edu

**Keywords:** ultrafast Raman spectroscopy, green fluorescent protein, single-site mutation, conformational inhomogeneity, excited state proton transfer, structural dynamics

## Abstract

Tracking vibrational motions during a photochemical or photophysical process has gained momentum, due to its sensitivity to the progression of reaction and change of environment. In this work, we implemented an advanced ultrafast vibrational technique, femtosecond-stimulated Raman spectroscopy (FSRS), to monitor the excited state structural evolution of an engineered green fluorescent protein (GFP) single-site mutant S205V. This mutation alters the original excited state proton transfer (ESPT) chain. By strategically tuning the Raman pump to different wavelengths (i.e., 801, 539, and 504 nm) to achieve pre-resonance with transient excited state electronic bands, the characteristic Raman modes of the excited protonated (A*) chromophore species and intermediate deprotonated (I*) species can be selectively monitored. The inhomogeneous distribution/population of A* species go through ESPT with a similar ~300 ps time constant, confirming that bridging a water molecule to protein residue T203 in the ESPT chain is the rate-limiting step. Some A* species undergo vibrational cooling through high-frequency motions on the ~190 ps time scale. At early times, a portion of the largely protonated A* species could also undergo vibrational cooling or return to the ground state with a ~80 ps time constant. On the photoproduct side, a ~1330 cm^−1^ delocalized motion is observed, with dispersive line shapes in both the Stokes and anti-Stokes FSRS with a pre-resonance Raman pump, which indicates strong vibronic coupling, as the mode could facilitate the I* species to reach a relatively stable state (e.g., the main fluorescent state) after conversion from A*. Our findings disentangle the contributions of various vibrational motions active during the ESPT reaction, and offer new structural dynamics insights into the fluorescence mechanisms of engineered GFPs and other analogous autofluorescent proteins.

## 1. Introduction

Vibrational frequencies of normal modes are the fingerprints of molecules. The accurate measurement and dynamic tracking of characteristic motions can offer valuable “bottom-up” insights into the conformation, structure, and evolution of the functional molecules of interest in the ground and excited state [1,2], and ideally in real time [3,4]. Among all the advanced molecular vibrational spectroscopic toolsets, femtosecond-stimulated Raman spectroscopy (FSRS) is a relative newcomer since the early 2000s and a promising structural dynamics technique that can simultaneously achieve high time resolution (<25 fs) and spectral resolution (<10 cm^−1^) in tracking photoinduced excited state (ES) processes [5,6,7,8,9,10]. Such a fundamental understanding of molecular transformations across non-equilibrium states has started to enable rational design of improved and new functions [10].

A typical FSRS setup consists of three incident laser pulses: a femtosecond (fs) actinic pump to initiate photochemistry, followed by a picosecond (ps) Raman pump and a broadband fs probe pair to monitor the excited state structural evolution [6]. As a photochemical reaction proceeds, the excited state electronic features often exhibit interesting dynamics during consumption of the reactant and formation of the product. Recent studies have shown that by taking advantage of the Raman resonance enhancement effect and wavelength tunability of the laser pulses, tuning the Raman pump to strategic wavelength locations can selectively and effectively probe certain transient species during a chemical reaction or certain subpopulations of the inhomogeneous distributions of molecules in solution [11,12,13].

FSRS has been successfully implemented to investigate a wide array of photosensitive systems, such as rhodopsins [3,14,15], phytochrome [16], myoglobin [17], blue-light photoreceptors using flavin [18,19], fluorescent proteins (FPs), and FP-based biosensors [4,20,21,22,23,24,25,26], photomolecular rotors [27,28], and photoacids [12,29,30,31,32]. Among them, wild-type green fluorescent protein (wtGFP) is a prototypical molecular machine [33,34]. This famous protein has a large fluorescent quantum yield (0.8) and Stokes shift (>5000 cm^−1^), which are due to its unique β-barrel structure and excited state proton transfer (ESPT) property from the embedded chromophore to its surroundings [35,36,37]. Its three-residue chromophore, with the Ser–Tyr–Gly (SYG) sequence, is in the protonated form (A) at ground state, but the proton at the phenolic end could be detached upon photoexcitation with UV light and, as a result, the chromophore reaches an intermediate deprotonated state (I*). In 2009, the Mathies group implemented FSRS with an 800 nm Raman pump to study this process, and discovered that several vibrational motions of the protonated chromophore in the singlet excited state (A*) exhibit prominent frequency and intensity oscillations [4]. Based on a detailed analysis of the period, phase, magnitude, and correlation of those quantum beats, they uncovered a ~120 cm^−1^ phenol ring wagging motion of the wtGFP chromophore, which likely gates the ESPT reaction. However, only two I* modes were observed in the conventional FSRS spectra (i.e., Raman pump at 800 nm), due to the decent resonance enhancement in A* state, but loss of resonance condition in the I* state.

In this work, we implemented the wavelength-tunable FSRS to study an engineered single-site mutant of wtGFP, GFP-S205V, which consists of the same SYG chromophore [38]. Since Ser205 is the first protein residue that connects to a conserved water molecule along the H-bonding chain from one side of the chromophore to the other side, a change to Val205 with no sidechain hydroxyl should block ESPT. However, green fluorescence was observed after exciting the A species at ~400 nm, so ESPT still occurs in the GFP-S205V mutant, but at a ~30-fold slower rate than wtGFP [4,35,38]. Based on the crystal structure of the mutant (PDB ID 2QLE) showing a reoriented Thr203 that comes for the rescue of an H-bonding chain hence ESPT reaction [38], as well as the all-atom explicit molecular dynamics (MD) simulations that predicted at least two conformations for Thr203 with fluctuating bulk water molecules in and out of the H-bonding chain [39], we use the newly developed tunable FSRS technique to answer the following questions. First, we aim to elucidate the key I* vibrational motions of the SYG chromophore that accompany and possibly facilitate the ESPT process in the engineered protein mutant. Second, we aim to study the photochemical reaction in a different environment from wtGFP, since the S205V mutation alters the local hydrogen(H)-bonding network around the chromophore. Third, we aim to monitor different subpopulations of this protein mutant in solution, in the ground and excited state, and unravel the dynamics prior to fluorescence. Furthermore, with critical comparisons to the SYG chromophore in wtGFP [4], a GFP-based emission ratiometric calcium ion biosensor called GEM-GECO1 [21], and an excitation ratiometric biosensor called GEX-GECO1 [24], as well as the TYG chromophore in another GFP-based intensiometric calcium biosensor called G-GECO1.1 [11,13], we will systematically analyze the pros/cons and similarities/differences of tuning the Raman pump to various wavelength locations to monitor the excited state structural dynamics of biomolecular systems. Our results offer important guiding principles to track key vibrational motions from the photoreactant to photoproduct species by strategically choosing the Raman pump wavelengths, as well as the Raman probe region in both Stokes and anti-Stokes FSRS. The mechanistic understanding of detailed structural dynamics pathways prior to fluorescence is directly applicable for the biosensor development and bioimaging applications by fluorescent protein engineers, biophysicists and biochemists, and life scientists.

## 2. Results and Discussions

### 2.1. Steady-State Electronic Spectra of the GFP-S205V Mutant

**Figure 1:**
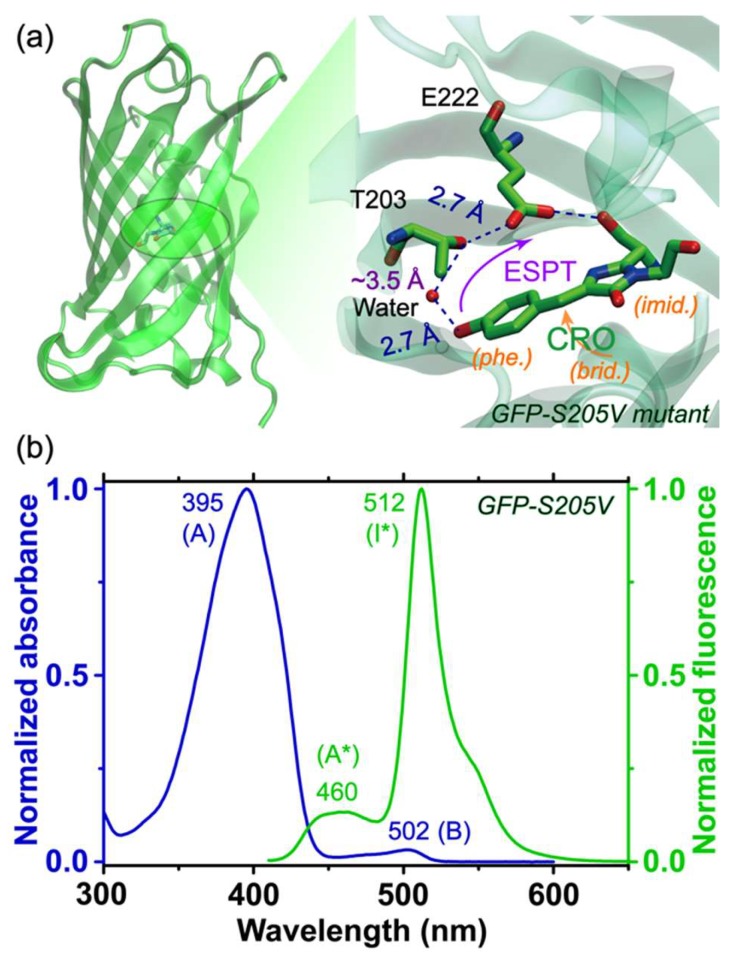
Schematic structure and electronic spectroscopy of the GFP-S205V mutant. (**a**) The GFP-S205V structure (PDB ID 2QLE) [38] and the chromophore (CRO) local H-bonding network. The CRO consists of a phenolic ring (*phe.*, labeled in orange) and an imidazolinone ring (*imid.*, labeled), linked by an ethylenic central bridge (*brid.*, labeled). The average O···O distances between H-bonding partners along the excited state proton transfer (ESPT) chain from multiple conformations are shown in angstrom units. The direction for ESPT reaction is shown by the magenta curved arrow. (**b**) Normalized absorbance (blue) and fluorescence (green) spectra under 400 nm excitation of GFP-S205V in aqueous buffer solution (pH = 7.9) at room temperature. The peak wavelengths and associated electronic state labels for the protein sample are denoted.

The chemical structure and chromophore local H-bonding network of the GFP-S205V mutant are shown in Figure 1a. The protein adopts a similar β-barrel structure to wtGFP, which can effectively shield the center chromophore (CRO) from the surrounding solvent. The proton at the phenolic end of the chromophore can be transferred to E222 along the H-bonding wire (CRO–water–T203–E222, which represents an alternative ESPT pathway from the highly efficient CRO–water–S205–E222 chain in wtGFP) upon 400 nm irradiation. This process has been investigated by time-resolved fluorescence, time-resolved IR, and molecular dynamics simulations, and it occurs on the hundreds of ps time scale with a kinetic isotope effect (KIE) of ~4, which is close to the corresponding value of ~5 in wtGFP [38,39,40]. The distance between a bridging water molecule and T203 was found to be appreciably lengthened from that in wtGFP, hence with reduced interactions to undergo the ESPT reaction [38]. However, the accurate ESPT time constant remains debatable from ca. 300 to 760 ps, depending on the ultrafast spectroscopies being used, and the data analysis methods [38,40]. In addition, detailed information about the participating vibrational motions from low- to high-frequency region of the protonated and deprotonated chromophore during the ESPT reaction is largely absent in literature.

As displayed in Figure 1b, the GFP-S205V mutant has a dominant absorption peak at 395 nm, attributed to its protonated form A [4,38]. The minor absorption at 502 nm is originated from the deprotonated B state population which reaches the thermal equilibrium with the A state population. A relevant time constant concerns the ground state proton transfer from I (the unrelaxed form of B) back to the A state with a ~400 ps time constant in water [37,41], whereas an earlier spectral hole-burning experiment showed that the photoinduced B reverting to A at thermal equilibrium could take hours [42]. Therefore, such thermally activated processes do not affect the ultrafast spectroscopy conducted in this work which focuses on transient excited state processes. Upon 400 nm photoexcitation of the A species, the emission spectrum shows a main peak at 512 nm with a bluer shoulder centered at 460 nm, which arises from the deprotonated chromophore intermediate excited state (I*) and the protonated chromophore excited state (A*), respectively. In addition, a deprotonated chromophore in a more relaxed environment (B*) can be accessed by direct excitation of the B species at ~500 nm [35].

### 2.2. Femtosecond Transient Absorption Monitors Electronic State Population Dynamics

**Figure 2:**
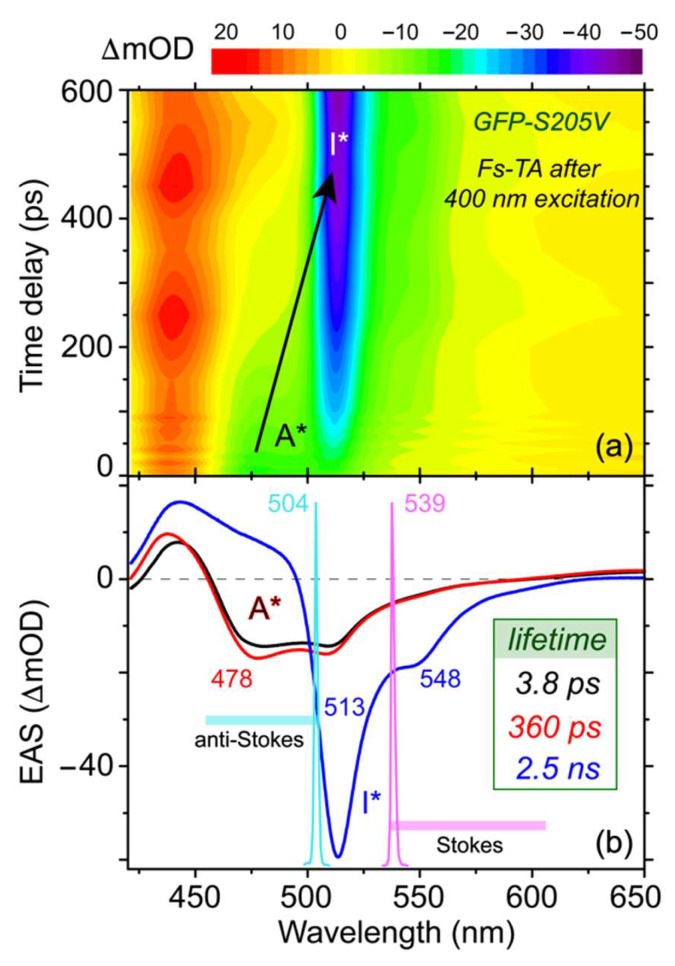
Femtosecond transient absorption (fs-TA) analysis implies the ESPT reaction mechanism inside the GFP-S205V mutant. (**a**) TA contour plot with transient electronic states labeled along the ESPT reaction. (**b**) Evolution-associated spectra (EAS) of the GFP-S205V mutant using a sequential kinetic scheme in global analysis. The lifetimes of the retrieved three EAS components are 3.8 ps (black), 360 ps (red), and 2.5 ns (blue). Peak wavelengths of the key TA features are labeled, and an I* state SE shoulder peak at ~548 nm is visible. The ps narrowband Raman pump pulses used are shown by the narrow spikes at 504 nm (cyan) and 539 nm (magenta), while the associated fs broadband Raman probe pulses are depicted by the color-coded horizontal bars either on the anti-Stokes (cyan) or Stokes (magenta) side.

To delineate the electron movements and charge transfer of GFP-S205V mutant in the excited state and also to help select the Raman pump tuning range, we first performed femtosecond transient absorption (fs-TA) spectroscopy on this protein mutant [43]. The 2D contour plot of the TA spectra from 420 to 650 nm is shown in Figure 2a. The spectra mainly consist of three regions: an excited state absorption (ESA) band below 460 nm, two stimulated emission (SE) bands (centered at ~478 and 513 nm) from 460 to 620 nm, and a broad ESA band above 620 nm. The SE band at 478 nm and the ESA band above 620 nm are both attributed to the reactant A* species [4,11], while the SE band at 513 nm is mainly from the product I* species. With progression of the photochemical reaction as the chromophore phenolic proton gets transferred in the protein interior, the SE band at 478 nm and ESA band above 620 nm gradually decay away, while the SE band at 513 nm gains intensity, indicating a two-state A*→I* transition after photoexcitation.

To uncover the molecular evolution during a photochemical reaction where the broad electronic features commonly overlap with each other, we performed global analysis using the Glotaran software with a sequential model and resolved three main stages [44]. The kinetic model for transient molecular species and the match between experimental and fitted traces across the spectral detection window at four representative time delay points are shown in Figure S1 (see the Supplementary Materials). Previous studies on related proteins have shown that a purely concerted model does not fit the TA spectra well, and some preparation stages typically exist before the main ESPT reaction step [21,26]. The resultant evolution-associated spectra (EAS) are plotted in Figure 2b with a root-mean-square (RMS) value of 1.318 × 10^−3^. Moreover, a four-component sequential kinetic model in global analysis leads to a ~13 fs initial component that lacks physical meaning. The resultant RMS is 1.309 × 10^−3^, which represents a mere ~0.7% improvement from the aforementioned three-component fit, indicating that the three-component model is sufficient in analyzing the fs-TA data in this work. The earliest EAS shows a 478 nm emission peak and a 442 nm ESA band, which are associated with the A* species that promptly emerge upon actinic photoexcitation. The small emission peak at 512 nm is likely from the fluorescence background (because it is present to a certain extent at negative time delay points) or some ultrafast ESPT channel via those pre-existing, largely optimal H-bonding chains [29,32,45]. The initial EAS then evolves to the second EAS with a time constant of 3.8 ps, during which the intensity of the A* SE band near 478 nm increases, indicating the necessity of a preparation stage before the ESPT process [4,21,26,46]. The ESA band below 450 nm also blue shifts, suggesting that the A* S_1_ state is stabilized on this time scale. Judging from the TA profile of A* species before the main ESPT step, the initial two EAS can be attributed to a well-defined A* state with a notable slope out of the Franck–Condon (FC) region, wherein the chromophore remains protonated. Reminiscent of a previously proposed stepwise-concerted-hybrid ESPT reaction mechanism in a GFP-S65T/S205V double mutant [26], the initial ~3.8 ps process likely involves some small-scale proton motions in close proximity to the chromophore (e.g., CRO···water, see Figure 1a) and a charge transfer (CT) step [26,32,47] in the partially deprotonated chromophore, which presumably set up the stage for the subsequent main ESPT step on the hundreds of ps time scale (see below).

The progression of the second EAS manifests the most drastic change with disappearance of the 478 nm SE band (A*) and growth of the 513 nm SE band (I*), corroborated by the steady-state fluorescence peak positions at ~460 and 512 nm (see Figure 1b). The retrieved time constant of this electronic population transition is 360 ps. Since the green fluorescence quantum yield of the GFP-S205V mutant is 0.78 [38], which is very close to wtGFP (0.80) that undergoes efficient ESPT on the 5–10 ps time scale [4,35,37], this 360 ps time constant can be mainly attributed to a lengthened ESPT process on ultrafast time scales, which does not negatively impact the overall fluorescence outcome in the fully equilibrated state. The third EAS then diminishes with a 2.5 ns time constant, largely in accord with the kinetic parameter of 0.31 ns^−1^ previously retrieved by fitting the time-resolved fluorescence data for both A* (at 450 nm) and I* (at 510 nm) after 388 nm excitation [38]. This component reflects a typical fluorescence process that depletes the excited state population [4,35,38].

For comparison, we performed target analysis with A* evolving into two excited state species in parallel, namely A*’ and I*, which decay away (i.e., back to the ground state). The same time constants of 3.8 ps, 360 ps, and 2.5 ns (like those in Figure 2b) were obtained which correspond to the lifetime of A*, A*’, and I*, respectively. Since the ESPT reaction (A*→I*) in this GFP-S205V mutant has been reported to be on the hundreds of ps time scale [38,40], the 360 ps lifetime should be associated with a deprotonation process of the chromophore (i.e., not just a relaxation within the protonated state), rendering this parallel model inconsistent with the main photochemical reaction pathway.

Based on the broad yet clearly assigned excited state electronic features of this protein mutant, three Raman pump wavelengths were carefully chosen in this study. First, an 800 nm Raman pump (not shown in Figure 2b that displays a spectral region up to 650 nm) is selected, which is pre-resonant with the A* broad ESA band above 620 nm. This Raman pump is suitable to track the A* electronic population dynamics, and has proven to be highly effective in studying GFPs and the GFP-based Ca^2+^-biosensors [4,11,21,24]. The other reason to choose this wavelength is that an 800 nm Raman pump is readily achievable in an optical setup from a commercially available laser amplifier system with an 800 nm fundamental pulse output (see Section 3.2 below). However, this wavelength is not suitable to track the I* species because they have no ESA or SE bands in this region (e.g., see the blue trace in Figure 2b). The second Raman pump we selected is at 539 nm (magenta spike in Figure 2b), which can achieve pre- or quasi-resonance (but not on resonance) with the broad I* SE band at 513 nm, capable of enhancing the I* features, as demonstrated before for similar protein systems [11,23]. In both cases, the Stokes FSRS was performed while the Raman probe wavelength (ca. 820–955 nm or 545–605 nm) is longer than the Raman pump (i.e., 801 nm or 539 nm). The third Raman pump we picked is at 504 nm (cyan spike in Figure 2b), with the Raman probe now located on the blue side (455–500 nm, i.e., anti-Stokes FSRS) [31,32,48]. Under this condition, the Raman pump is pre-resonant with the A* SE band at 478 nm, while the probe better overlaps with this SE band [32] without being absorbed by the A ground state species near 400 nm. Therefore, strong Raman signal of the transient A* species with sufficiently high signal-to-noise ratio (SNR) can be achieved on ultrafast time scales.

### 2.3. Ground State Raman Spectra with Different Raman Pumps Provide Insightful Comparisons

**Figure 3:**
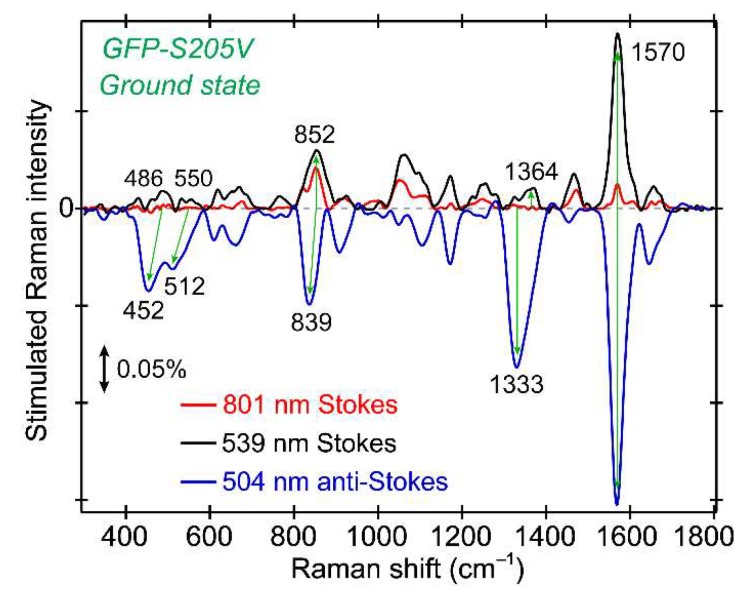
Ground state femtosecond-stimulated Raman spectroscopy (FSRS) of the GFP-S205V mutant with 801 nm (red), 539 nm (black), and 504 nm (blue) Raman pump in the Stokes, Stokes, and anti-Stokes experimental setup, respectively. The double-arrowed line denotes the stimulated Raman gain magnitude of 0.05%. The dashed gray line marks zero Raman gain. The green arrows highlight some marker band intensity and frequency change from the conventional FSRS (red) to tunable FSRS (black and blue traces).

To benchmark the chromophore vibrational modes, the ground state (GS) FSRS spectra of the GFP-S205V mutant are plotted in Figure 3, which make use of the Raman pump–probe pair without the photoexcitation pulse. Most of the Raman peaks appear at similar locations regardless of the Raman pump wavelengths, validating their association with the photosensitive chromophore. However, the mode intensity increases dramatically as we tune the Raman pump closer to resonance conditions with ground state absorption of the protein chromophore (see Figure 1b). As seen for the ~1570 cm^−1^ mode, it is significantly enhanced as the Raman pump changes from 801 to 504 nm, approaching the B (A) absorption peak at 502 (395) nm. Besides the increased intensity, an important benefit of performing FSRS using a Raman pump not at 800 nm is the higher sensitivity to detect low-frequency motions. Since the laser fundamental output was used to generate the supercontinuum white light as the near-IR Raman probe, the strong incident 800 nm light cannot be completely filtered or eliminated from the resultant weak white light (see Section 3.2 below for details). As a result, the 800 nm residual in Raman probe can interfere with the 800 nm Raman pump and contaminate the low-frequency vibrational region (e.g., 802–820 nm, which corresponds to vibrational modes from ~30–300 cm^−1^) [4,21]. Moreover, the lowest frequency we observed for a fluorescent protein sample is ~400 cm^−1^ [22], which is likely due to the scattering issues intrinsic to protein size. For comparison, a smaller organic chromophore, like a photoacid in solution, allows the observation of low-frequency modes below 300 cm^−1^, such as the ~180 cm^−1^ H-bond stretching mode for pyranine in water [12]. In a nice contrast, tuning the Raman pump to the visible region allows the use of broadband Raman probe photons away from 800 nm which enables a much better observation of the low-frequency motions below ~600 cm^−1^ [10,12]. The direct observation of multiple vibrational modes across a broad spectral window (typically over 1500 cm^−1^) is a notable advantage of FSRS over other ultrafast spectroscopic techniques. To observe even lower frequency modes (e.g., <300 cm^−1^) for protein samples in solution, which may report slower protein motions or collective (likely intermolecular in nature) vibrational motions that are coupled to the ultrafast processes such as ESPT reaction, other advanced spectroscopic techniques such as the femtosecond time-domain Raman spectroscopy [25] and terahertz absorption spectroscopy [49,50] can be implemented.

When comparing the GS peaks collected by the Stokes FSRS (with 539 nm Raman pump and 545–605 nm Raman probe) and the anti-Stokes FSRS (with 504 nm Raman pump and 455–500 nm Raman probe), it becomes clear that some of the vibrational motions exhibit frequency red shifts. For example, the 852 cm^−1^ mode shows an intensity increase without frequency change as the Raman pump is tuned from 801 to 539 nm in the Stokes FSRS, however, the mode not only gains further intensity but also shifts its frequency to 839 cm^−1^ in the anti-Stokes FSRS with a bluer Raman pump at 504 nm. Similarly, two lower frequency modes at 486 and 550 cm^−1^ also exhibit stronger intensity and notable red shifts. This observation substantiates the improved resonance Raman enhancement when both the Raman pump and probe wavelengths better match an electronic absorption band [51,52] and, in this particular case, the deprotonated chromophore Raman signal gets enhanced due to its absorption peak at ~502 nm (Figure 1b). Such a Raman pump-dependent FSRS peak center frequency in association with different molecular subpopulations of an inhomogeneous sample has been demonstrated [10,13,53]. Moreover, some excited state vibrational coherences (typically broader peaks) may be generated when the Raman pump effectively overlaps with the absorption band of a certain species [31,54]. Further comparisons to the excited state FSRS can be found below in Section 2.5, Section 2.6 and Section 2.7, and these intriguing mode frequency red shifts in Figure 3, associated with the enhancement of deprotonated chromophore species, can corroborate the transient Raman mode assignment during the ESPT reaction (see below). The essence of performing time-resolved FSRS is to capture transient Raman modes in the excited state before the system returns to the electronic ground state [7,10], so the resonance conditions of the Raman pump–probe pair need to be carefully selected to avoid generating significant ground and excited state species simultaneously (which could lead to spectral overlap and dispersive line shapes [31,52]). Fortunately, pre-resonance conditions have been experimentally validated to track excited state vibrational dynamics during a photochemical reaction, such as ESPT in solution [12,32] or protein environment [8,11,13,23,26], also showing apparent vibrational frequency shifts from the ground to excited state (e.g., S_0_→S_1_) of the chromophore.

### 2.4. Stokes FSRS with an 801 nm Raman Pump Benchmarks A* Vibrational Dynamics

**Figure 4:**
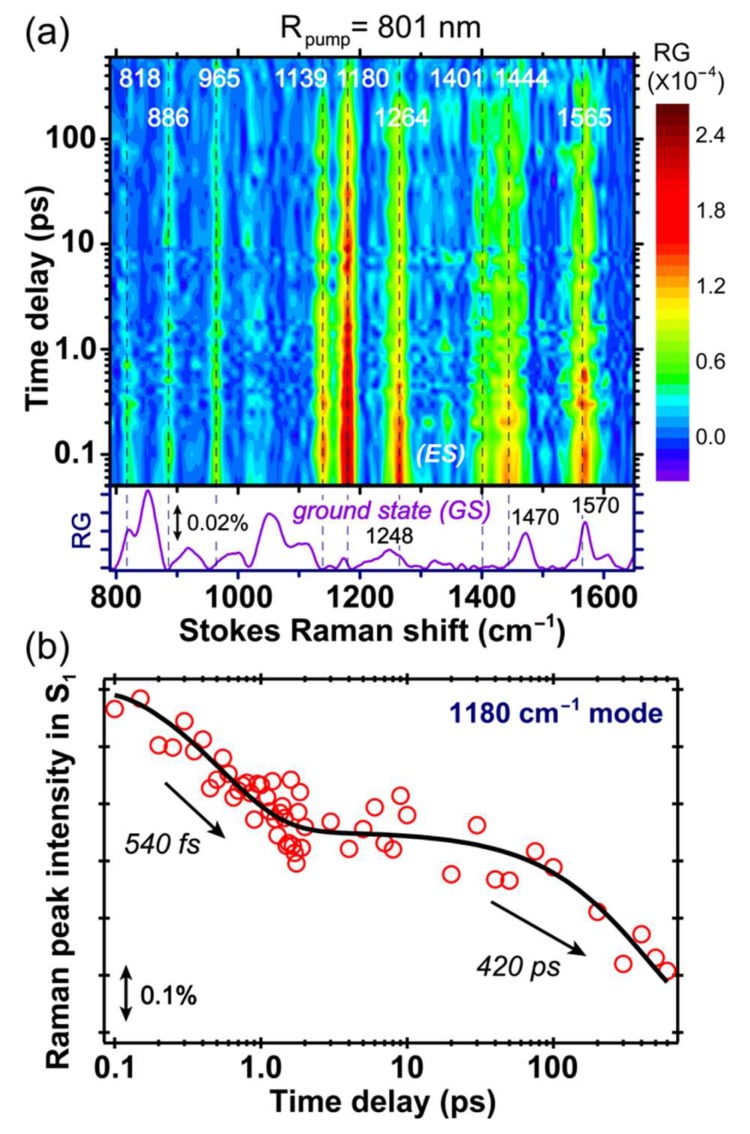
Time-resolved FSRS with an 801 nm Raman pump elucidates the A* structural evolution. (**a**) Semilogarithmic contour plot of the Stokes FSRS spectra of the GFP-S205V mutant after 400 nm photoexcitation. The photoinduced vibrational mode frequency shift and intensity change from S_0_ into S_1_ are highlighted by the vertical dashed lines. (**b**) Semilogarithmic plot of the A* 1180 cm^−1^ marker band intensity decay (data points shown in red circles) with the fit in black solid curve. The least-squares fitted time constants are denoted by the respective arrows. The stimulated Raman gain magnitude of 0.1% is depicted by the double-arrowed vertical line.

The excited state FSRS spectra of GFP-S205V were first collected with an 801 nm Raman pump at the Stokes side. The 2D contour plot from ca. 800 to 1650 cm^−1^ and up to 600 ps after photoexcitation is presented in Figure 4a. Note that the ground state spectrum is subtracted from all the experimentally collected excited state spectrum, so the resultant difference spectrum at each time delay point after photoexcitation corresponds to the “pure” excited state species [4,7]. Further verification of the excited state mode dominance in our data analysis comes from the observed dynamics and distinct frequencies/line shapes in a non-equilibrium state (versus the electronic ground state), and the broad peak width that does not significantly vary starting from photoexcitation time zero on the relevant time scale for the photochemical reaction under study (without a significant internal conversion pathway back to a hot ground state) [4,8,10,12]. All the transient modes reach their maximal intensities within the cross-correlation time (~140 fs) between the fs actinic pump and Raman probe, followed by decay on the hundreds of ps time scale. Based on their intensity dynamics, these motions are attributed to the reactant species A* with a protonated chromophore in S_1_, because the modes from the product I* species should mainly rise on the hundreds of ps time scale as the ESPT reaction occurs inside the protein [11,21].

**Figure 5:**
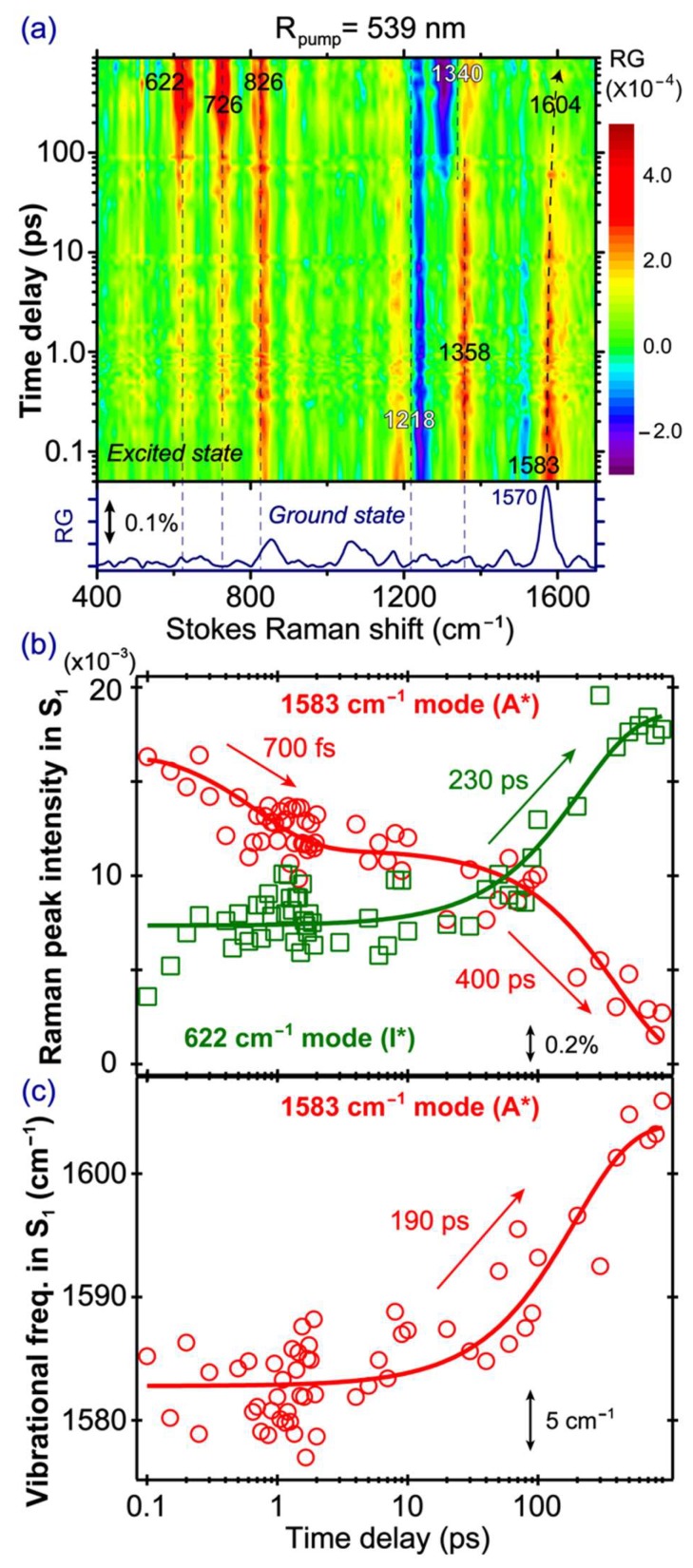
Tunable FSRS with a visible Raman pump unveils rich photochemistry. (**a**) Semilogarithmic contour plot of the Stokes FSRS time-resolved spectra of the GFP-S205V mutant with 539 nm Raman pump up to 900 ps after 400 nm photoexcitation. The ground state FSRS spectrum (blue) is plotted below for comparison. The dispersive line shapes are marked by the dashed lines in between the positive and negative components at ~1218 and 1340 cm^−1^. (**b**) The intensity dynamics of the A* 1583 cm^−1^ mode and I* 622 cm^−1^ mode in the electronic excited state. The time constants from least-squares fits are listed by the color-coded arrows on characteristic time scales. (**c**) The frequency dynamics of the 1583 cm^−1^ mode with the single exponential fit time constant.

**Figure 6:**
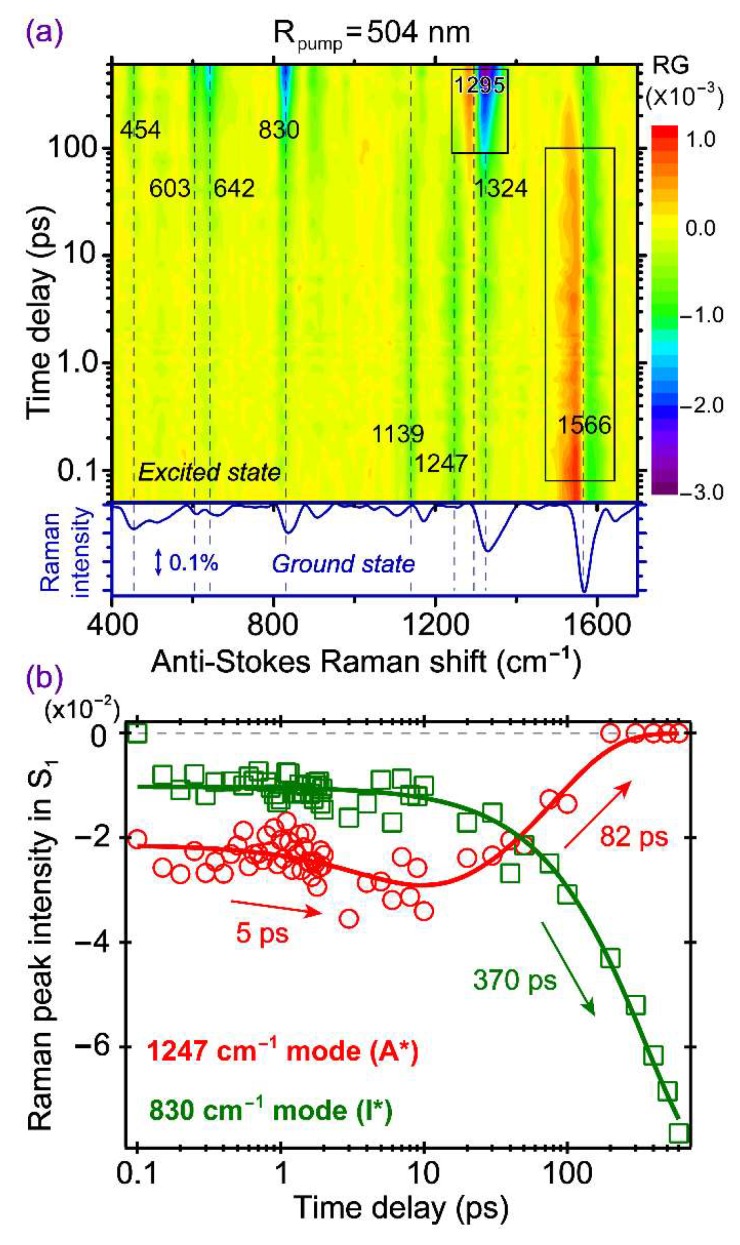
Anti-Stokes FSRS unveils deeper structural dynamics insights. (**a**) Semilogarithmic contour plot of anti-Stokes FSRS of the GFP-S205V mutant with 504 nm Raman pump after 400 nm photoexcitation. Transient vibrational marker bands are labeled. The black rectangles highlight the dispersive line shapes of A* and I* species around 1566 and 1295 cm^−1^ at early and late time delay points, respectively. The frequency differences between the ground and excited state Raman modes to various degrees are visible across the detection window (see vertical dashed lines). (**b**) Transient intensity dynamics of the A* 1247 cm^−1^ mode and the I* 830 cm^−1^ mode as Raman loss signals (i.e., negative peaks). The pertinent excited state time constants are denoted.

**Figure 7:**
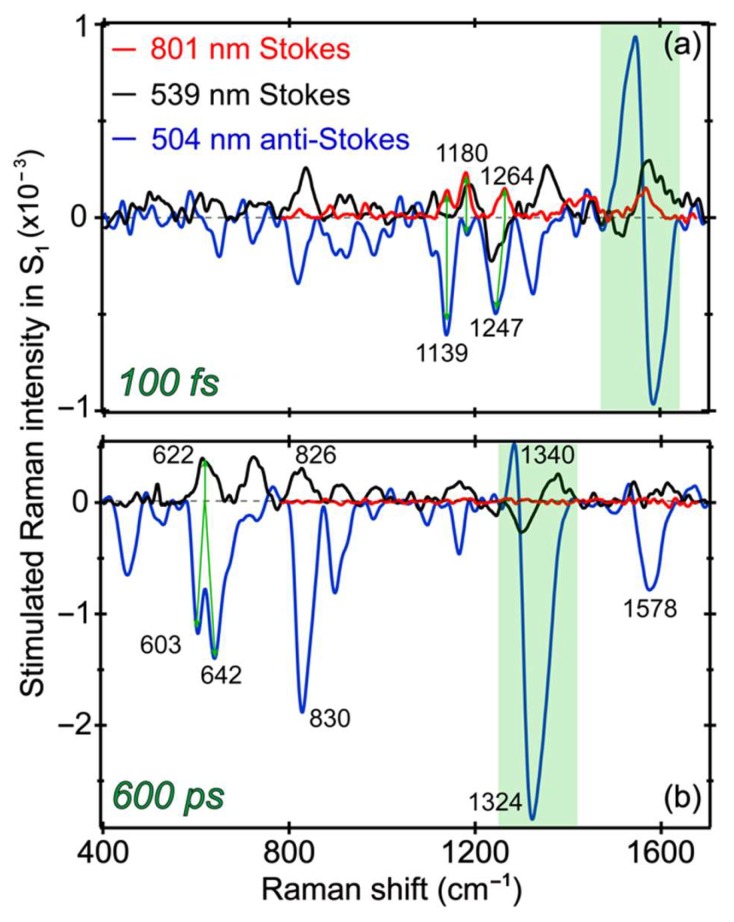
Overlaid excited state FSRS spectra of the GFP-S205V mutant with Raman pump at 801 nm (red), 539 nm (black), and 504 nm (blue) at the time delay of (**a**) 100 fs and (**b**) 600 ps, following 400 nm photoexcitation. The mode-dependent dynamic Raman line shapes are apparent, and a number of marker band frequencies are labeled. Green arrows highlight the relationships between a few Raman mode frequencies as the Raman pump is tuned, and the semi-transparent green shades emphasize the dispersive line shapes around ~1565 and 1330 cm^−1^ in (**a**,**b**), which are likely associated with the excited state A* and I* species of the chromophore, respectively.

In Figure 4b, we plotted the intensity dynamics of the A* 1180 cm^−1^ mode. This mode consists of the chromophore phenol ring-H and bridge C–H rocking motion, and is a sensitive probe to track the ESPT process based on our former studies [11,13,21,24]. The mode intensity plot on a linear scale, from negative time points to 10 ps after photoexcitation to the A* S_1_ state, is shown in Figure S2 in the Supplementary Materials, and the signal rise around time zero within the cross-correlation time (~140 fs) is clearly visible. A semilogarithmic plot across a much wider detection time window (~600 ps) is displayed in Figure 4b to better represent the excited state signal decay, and its vibrational intensity dynamics can be fit with a biexponential decay with ~540 fs and 420 ps time constants. The first time constant is likely due to the photoexcited chromophore moving out of the FC region, along with adjacent solvent molecular motions, and since a rapid change of the energy gap between electronic states (e.g., S_1_–S_2_, S_1_–S_0_) is expected, the resonance condition for the transient A* species changes along with the electric polarizability of the chromophore in its dynamic, rapidly fluctuating local environment. The second time constant is similar to the 360 ps retrieved from global analysis of the fs-TA data in Figure 2b, therefore confirming the time scale for ESPT reaction. It is notable that the Raman mode coherences, as shown in Figure 4, Figure 5, Figure 6 and Figure 7 (see below), are generated by the ps-Raman-pump and fs-Raman-probe pair, at well-defined time delay points after actinic photoexcitation [6,8,10], so these vibrational coherences effectively act as an array of “probing” modes, to capture the conformational snapshots at those particular time points and their associated transient electronic state population. In this context, the similarity between time constants of 360 and 420 ps in Figure 2b and Figure 4b supports the 1180 cm^−1^ mode assignment to the A* state, while the larger time constant from time-resolved FSRS may arise from (1) some spectral overlap with the neighboring vibrational modes, (2) spectral overlap in TA that leads to some uncertainties in retrieving time constants from global analysis, and (3) resonance Raman condition change for the A* modes during the A*→I* transition [31,52].

Since the initial energy relaxation has occurred within ~2 ps as shown in Figure 4b (i.e., the early-time inflection point on the A* marker band intensity dynamics fitting curve), this clear separation of two characteristic time constants reveals an activation energy barrier for ESPT as the A*→I* transition becomes the rate-limiting step for A* Raman mode decay, as well as the I* mode rise. The latter process represents a more direct link because ESPT is the only channel generating I* species, whereas it is one of the pathways depleting A* species.

Notably, a comparison between the FSRS spectra of the GFP-S205V mutant and wtGFP with the same 800 nm Raman pump [4] shows that all the excited state vibrational mode frequencies are largely conserved, as well as the instantaneous mode frequency shift around photoexcitation time zero (see the vertical dashed lines in Figure 4a) due to electron redistribution upon 400 nm electronic (π→π*) excitation of the A species. These spectral changes upon photoexcitation are deeply rooted in the photoacid nature of the protein chromophore, and the rapid protonation state change in the excited state [4,12,21] as the overall electron density migrates from the phenolic ring to the imidazolinone ring (i.e., charge transfer that occurs on ultrafast time scale). However, the dynamics of Raman marker bands in the GFP-S205V mutant are much longer than wtGFP, which match the ESPT time constant obtained by other ultrafast spectroscopic techniques [38,40]. This result not only benchmarks the characteristic A* modes of an embedded SYG chromophore, but also proves the sensitivity and reliability of using FSRS to track an array of vibrational motions and monitor a photochemical reaction such as ESPT.

### 2.5. Stokes FSRS with a 539 nm Raman Pump Tracks I* Vibrational Dynamics

To unravel the characteristic I* vibrational dynamics of the SYG chromophore, we tuned our Raman pump at 539 nm to be pre-resonant with the SE band of the emerging I* species (Figure 2b), and Figure 5a presents the time-resolved 2D contour plot of the FSRS spectra of the GFP-S205V mutant. In contrast to the abovementioned FSRS spectra with an 800 nm Raman pump, major differences upon tuning the Raman pump to 539 nm are that some pronounced modes (622, 726, and 826 cm^−1^) reach their maximal intensities after several hundreds of ps, not at time zero. This phenomenon infers the I* nature of these modes, and confirms that tuning the Raman pump toward particular electronic bands can effectively enhance and detect the vibrational features of transient molecular species. In addition to the I* modes with largely absorptive line shapes (e.g., positive peaks that have a gaussian profile for these chromophore modes inside a protein pocket), we also observe a dispersive line shape at ~1218 cm^−1^ (the negative portion peaks at ~1241 cm^−1^) at early times when the chromophore is mainly in the A* state. This dispersive line shape diminishes with time, while a new dispersive feature appears at ~1340 cm^−1^. This rather unusual observation indicates that the dispersive line shape still allows the dynamic tracking of a photochemical reaction. The 1241 cm^−1^ mode could be strongly coupled with the A* electronic transition to a nearby state (e.g., S_1_→S_0_), while the 1340 cm^−1^ mode is coupled with an I* electronic transition (e.g., S_1_→S_0_). Such a mode-dependent line shape change due to vibronic transitions in Stokes FSRS has been reported for photoexcited chromophores in solution [18,52], especially on or near resonance conditions. We conjecture an intricate dependence of the A* Raman peak line shape on the resonance conditions with respect to overlapping electronic bands at early times (Figure 2b). Since the evolving A* SE band and ESA band likely overlap in the spectral region of the Raman pump and probe we used in this case (i.e., 539 nm and ~545–605 nm), we observed clear dispersive line shapes at certain Raman marker bands which undergo vibronic transitions in a mode-specific manner during the FSRS signal generation [52,55].

With this tunable FSRS dataset, the aforementioned Raman mode frequency red shift in Figure 3 can be further rationalized. In the low-frequency region, the ground state 852 cm^−1^ mode appears at 839 cm^−1^ when the Raman pump was tuned from 539 to 504 nm, indicating that when the deprotonated chromophore (B species in the ground state) gets further enhanced, a lower frequency is observed. The related I* species, when accumulated in time, exhibit the mode at 826 cm^−1^ (see Figure 5a). Similarly for the high-frequency mode at 1364 and 1333 cm^−1^ with the 539 and 504 nm Raman pump, respectively, the nearby I* mode is at ~1340 cm^−1^ (Figure 5a). In essence, the deprotonated chromophore exhibits these characteristic lower frequencies of Raman modes due to change of the conjugation structure, as well as the overall energy stabilization achieved by a stronger H-bonding network surrounding the photoacidic chromophore [12,56]. The general consideration to enhance the I* species is thus to use a Raman pump in the bluer wavelength region, but away from the SE peak.

In Figure 5b, the 1583 cm^−1^ A* mode decay and the 622 cm^−1^ I* mode rise are overlaid and correlated in one graph (see the mode assignments in Table 1 below). The 1583 cm^−1^ mode is mainly a C=N stretch mixed with some C=C and C=O stretching motions in the excited state [4,40]. It appears at a higher frequency than the 1565 cm^−1^ mode observed in the 800 nm Raman pump FSRS (Figure 4a), suggesting that different Raman pumps can enhance different A* species [13]. We consider that different resonance conditions achieved by the Raman pump pulse cannot directly lead to the observed Raman mode frequency variation because the vibrational energy gaps within the same electronic state remain the same, regardless of the Raman pump wavelength being used [13]. In particular, the 539 nm Raman pump likely enhances a slightly more deprotonated A* population, because in those chromophores with different H-bonding configurations from the mainly protonated A* population, more electrons are distributed across the phenol and imidazolinone rings, and can cause the frequencies of certain mixed C=N, C=C, and C=O stretching motions to blue shift [13,23]. For example, a higher frequency of the C=N stretching motion is expected when charge transfer occurs from the phenolate end to the imidazolinone end in a deprotonated SYG chromophore, which is characteristic of a photoacid that undergoes ESPT in solution or protein matrix [26,32]. The 1583 cm^−1^ mode decays biexponentially with the 700 fs and 400 ps time constants. The similarity of the time constants between this A* mode and the 1180 cm^−1^ A* mode in conventional FSRS (800 nm Raman pump, see Figure 4b) indicates that although the two Raman pumps allow the probing of different A* subpopulations, their intensity decay dynamics remain largely unchanged, which we attribute to the large O···O distance between the bridging water and T203 (i.e., ~3.5 Å; the three subpopulations from MD simulations show the dynamic distance distribution peaks at ~2.75, 4.0, and 4.5 Å) in the ESPT chain that is the rate-determining step for ESPT (see Figure 1a) [38,39]. This represents the reaction “bottleneck” as infrequent proton transfer occurs between these key players in the ESPT chain. By contrast, the distance observed from crystal structures of wtGFP between the chromophore hydroxyl oxygen atom and the adjacent conserved water molecule is ~2.6 Å [57,58,59] which was supported by classical MD simulations and multiconfigurational electronic structure calculations [47], also matching that same distance in the GFP-S205V mutant at ~2.7 Å [38] (see Figure 1a).

Notably, the 622 cm^−1^ I* mode exhibits a faster rise with a ~230 ps time constant, when compared to the A* mode decay (~400 ps, Figure 5b). A closer examination of the rise dynamics of this I* mode shows that its intensity promptly rises upon photoexcitation, reaching a plateau from ca. 300 fs–10 ps, and then rises again on the hundreds of ps time scale. This observation could stem from a small A* subpopulation that undergoes proton dissociation rapidly upon photoexcitation, consistent with a pre-existing largely optimal H-bonding chain (see above, Figure 2b) as well as multiple conformations of key players, such as T203 and bridging water in the chain [29,32,39,45]. The mode intensity of the early-time-generated I* species accounts for ~39% of the overall I* intensity, which could slightly decrease the main ESPT time constant by fitting the later intensity rise of the mode. However, the clear trend of a smaller time constant for I* rise (~230 ps) than that for A* decay (~400 ps) is reasonable, because besides ESPT, there are other A* energy dissipation pathways that occur on longer time scales (e.g., non-radiative ring twisting, fluorescence) [11,21,46] or from some trapped A* species undergoing vibrational cooling, which effectively compete with the proton transfer kinetics. The ESPT time constant in the GFP-S205V mutant is therefore better reflected by the 230 ps time constant. To corroborate this new experimental observation, we performed a quantitative estimate of the proton transfer rate for S205V in water on the basis of a temperature-dependent study of the decay of A* blue fluorescence, which is slowed down upon lowering the temperature from 318 to 132 K [38,61]. Using the reported ESPT activation energy of $E_{a}$ ≈ 18.2 kJ/mol and an exponential prefactor A ≈ 6.7 × 10^12^ s^−1^ (corresponding to an intrinsic proton transfer rate of ~150 fs [62], which also matches a barrierless reaction rate constant approximately as the frequency factor $\left(k_{B}T/h\right)$ ≈ 6.1 × 10^12^ s^−1^), we used the Arrhenius equation $k=A\cdot exp(-E_{a}/RT)$ at room temperature (T≈295 K), and derived the ESPT reaction rate constant $k\approx 4.0\times 10^{9}$ s^−1^, which corresponds to a time constant of ~250 ps. This is clearly consistent with the 230 ps time constant we retrieved from the I* vibrational marker band intensity rise in the excited state FSRS experiment.

Furthermore, the 1583 cm^−1^ mode exhibits a notable frequency blue shift (1583→1604 cm^−1^) with time (Figure 5c), likely due to the vibrational cooling process in A*. This contrasts with the previously observed mode frequency red shift (1585→1565 cm^−1^) in wtGFP [4] and blue shift (1570→1584 cm^−1^) in the calcium biosensor GEM-GECO1-P377R mutant [23] with an 800 nm Raman pump, both closely tracking the ESPT reaction of the largely protonated SYG chromophore. Given the sudden influx of photoexcitation energy, the chromophore vibrational cooling through high-frequency motions can be an effective means for energy dissipation [24,63,64]. Similarly, in a small organic photoacid HPTS, its 1530 cm^−1^ mode (ring C=C stretching and phenolic COH rocking motion) from its excited photoacid state (PA*) also shows a frequency blue shift, and the shift magnitude increases with more energetic excitation photons [12,64]. Because of the spectral overlap in this high-frequency region, the mode frequency red shift due to photochemistry (associated with the A* species undergoing ESPT reaction to I*) and mode frequency blue shift due to photophysics (associated with the A* species undergoing vibrational cooling within A*) could mix, and exhibit some cancellation effect. In the current work with a different resonance condition achieved by the 539 nm Raman pump, due to the observed frequency blue shift (Figure 5c) and intensity decay (Figure 5b) of the 1583 cm^−1^ marker band, this mode is dominantly an A* mode, and the observed time constant of ~190 ps can thus be attributed to vibrational cooling in parallel with ESPT. Interestingly, we previously reported a vibrational cooling time constant of ~70 ps based on the 1265 cm^−1^ A* marker band for an embedded SYG chromophore in a calcium biosensor GEX-GECO1 using an 800 nm Raman pump [24]. The significant lengthening of this time constant (70→190 ps) could be a result of the GFP-S205V mutant having a well-protected chromophore at the center of the GFP β-barrel, while the Ca^2+^-free GEX-GECO1 has an opening on the circularly permuted GFP, so the lack of flexible water molecules and a more rigid environment in the GFP-S205V mutant results in a longer time for vibrational cooling and energy relaxation into the surroundings [31,65]. Moreover, the H-bonding network differs in the chromophore pocket of these two proteins. Larger interatomic distances between key players in the H-bonding chain (see Figure 1a), which significantly lengthen the ESPT time constant in the GFP-S205V mutant when compared to the GEX-GECO1 protein biosensor, could also be responsible for a lengthened vibrational cooling time constant for the A* species that remains in the same electronic state.

### 2.6. Anti-Stokes FSRS with 504 nm Raman Pump Provides Further Insights into ESPT and Other Pathways

To gain more information about the transient A* species by taking advantage of the A* SE band (see Figure 2b), we performed the anti-Stokes FSRS with a 504 nm Raman pump that locates to the red side of the main A* SE peak, while also to the blue side of the main I* SE peak. The wavelength selection adopts a balanced approach to track A* modes at early times and I* modes at later times, while avoiding an overlap with the bluer ground state bleaching band of A* species. This setup represents a rare case that anti-Stokes FSRS [10,20,48] has been applied to study proteins, and the choice of Raman pump wavelength is important in the information content being retrieved from such an experiment. The time-frequency 2D contour plot is presented in Figure 6, and most peaks are negative due to the FSRS signal generation pathways when the bluer Raman probe better matches the A* SE band than the redder Raman pump [32,66]. The 1139 and 1247 cm^−1^ modes are attributed to the A* species because they display the monotonic decay behavior. The 1566 cm^−1^ mode shows a very strong dispersive line shape at early time (see Figure S3a for the raw experimental spectrum at 100 fs time delay and the spline baseline drawn), which gradually decays with time, so it can also be attributed to an A* mode. The line shape eventually changes to a negative peak after ~300 ps (see Figure S3b for the raw experimental spectrum at 600 ps, and the spline baseline drawn), which implies that when the A* population decreases via ESPT and other relaxation pathways, there is likely an electronic feature change of the A* species, so the resonance condition worsens with the dispersive line shape diminished [52,66]. This is because the pertinent hot luminescence terms mainly responsible for such mode-dependent dispersive line shapes require the resonance or quasi-resonance condition to be favorable and maintained (i.e., the Raman pump and/or probe wavelength matching the transient electronic peak position associated with each mode). If the initially dominant dispersive line shape at ~1566 cm^−1^ is due to the overlap between the Raman pump and probe pair and the A* SE band around 478 nm (e.g., for observation of the 1566 cm^−1^ mode with the Raman pump at 504 nm, the corresponding Raman probe is at 467 nm), then, as the ESPT reaction progresses on the hundreds of ps time scale, the transient electronic band shifts to the I* SE band around 513 nm (Figure 2) that is farther away from the Raman probe. As a result, the reduction of hot luminescence terms leads to the apparent diminishment of dispersive line shapes for this mode (see Figure 6a and Figure S3b).

Notably, besides the prominent A* modes above 1100 cm^−1^ (including the one at 1324 cm^−1^ before ~50 ps), several I* modes are enhanced and clearly observed at later time points. We consider that although the Raman probe overlaps with the A* SE band at early times, the 504 nm Raman pump is still within the I* SE band. At later times, as ESPT reaction proceeds and generates a significant I* population, the Raman probe better overlaps with the I* ESA band that “takes over” the A* SE band below 500 nm (see Figure 2b). All these factors favor the improved resonance conditions for the photoproduct state and, consequently, increase the stimulated Raman signal intensity of the nascent I* species (mainly negative peaks, and one mode with dispersive line shape at ~1295 cm^−1^ in Figure 6a). Interestingly, the dispersive mode frequency largely matches the energy gap between the I* SE main peak at 513 nm and a vibronic transition shoulder at 548 nm (Figure 2b), corresponding to an excited state vibrational mode at ~1250 cm^−1^ that could be used as a sensitive vibrational probe for ESPT in the protein matrix.

In Figure 6b, the intensity dynamics of the 1247 cm^−1^ A* mode and the 830 cm^−1^ I* mode are displayed as representative vibrational probes for the ESPT reaction inside the GFP-S205V protein pocket based on the anti-Stokes FSRS measurement. The evolution of the 1247 cm^−1^ mode shows a dramatic difference from the dynamics of other A* modes observed in Stokes FSRS with the 801 nm (Figure 4) or 539 nm (Figure 5) Raman pump. This mode has a small but distinct rise phase, with a 5 ps time constant, which largely matches the transition time of ~4 ps from the first to second EAS in global analysis of the fs-TA spectra (Figure 2b). It is noteworthy that this A* marker band (assigned to the chromophore C–O stretching motion) appears at ~1264 cm^−1^ in Stokes FSRS with an 801 nm Raman pump (Figure 4a) and ~1218 cm^−1^ at the “zero crossing point” of a dispersive line shape [52] in Stokes FSRS with a 539 nm Raman pump (Figure 5a), and similar modes were observed for the SYG chromophore inside wtGFP [4] and the abovementioned GFP-derived calcium ion biosensor GEM-GECO1 [21]. Therefore, this key result corroborates the occurrence of an initial phase of protonated chromophore conformational motions which helps the A* species to evolve into a more stable state, likely better solvated by the surrounding water molecules and protein residues, leading to an increased A* SE intensity as well as a blue shift of the ESA band (Figure 2b). Notably, in a time-resolved fluorescence up-conversion experiment on GFP-S205V monitoring the A* emission at 470 nm [40], a ~9 ps component was observed in the A* fluorescence decay with 28% weight, preceded by a ~200 fs component that has 50% weight (solvation dynamics or vibrational relaxation could contribute) and followed by a ~250 ps component with 22% weight (consistent with the A*→I* ESPT time constant). Since the 9 ps step does not exhibit KIE and hence does not involve proton transfer, this temporal component could be related to the ~4 ps component recovered from our global analysis of the fs-TA data (Figure 2b), as well as the ~5 ps component retrieved from kinetic analysis of the transient Raman mode intensity (Figure 6b). The time-resolved fluorescence time constant is slightly longer than that from the time-resolved electronic dynamics, due to the stimulated nature of our fs-TA measurement [43,67].

Furthermore, the main reason we observed this distinct ~5 ps step prior to the main ESPT reaction is the unique resonance condition achieved by the 504 nm Raman pump and a bluer Raman probe, wherein the Raman probe essentially overlaps with the A* SE peak region. For example, the Raman probe generating the 1247 cm^−1^ mode is at 474 nm, which is very close to the A* SE peak at 478 nm. Our previous study on a similar resonance condition with the Raman pump pre-resonance but Raman probe on-resonance with respect to an electronic absorption band in the ground state for a fluorescent dye rhodamine 6G in methanol, also showed a non-dispersive mode line shape [66], which constitutes a useful strategy to track the high-frequency Raman modes of transient species with improved SNR while maintaining a Raman gain or loss line shape on the anti-Stokes side.

From the least-squares fit of the intensity dynamics plot in Figure 6b, the ~1247 cm^−1^ A* mode decays with an 82 ps time constant, much faster than the ~400 ps ESPT time constant. The nearby ~1139 cm^−1^ mode exhibits a similar dynamic plot with these time constants. A possible explanation is that this tens of ps process is related to the protein chromophore relaxation from A* to A. Interestingly, an earlier study of the same protein mutant using time-resolved IR spectroscopy and species-associated global analysis reported a ~38 ps process that was attributed to an A*→A nonradiative transition (e.g., intramolecular motions of the chromophore that promotes its internal conversion back to S_0_) [40]. Four reasons may explain why this peculiar process is observed in the anti-Stokes FSRS with a 504 nm Raman pump. First, the Raman probe overlaps with the A* SE band, and can sensitively track the structural dynamics of all the A* subpopulations, regardless of their ESPT capabilities. Second, this Raman mode exhibits a dispersive line shape in Stokes FSRS with a 539 nm Raman pump, indicative of its strong coupling to the A* downward transition (i.e., S_1_→S_0_). Third, at late time delay points, the positive component of the emerging I* dispersive peak (center at ~1295 cm^−1^ in Figure 6a) overlaps with the negative peak at ~1247 cm^−1^, resulting in an apparent intensity loss. Fourth, even if the pertinent A*→A transition is actually a vibrational relaxation process within A*, and the Raman intensity decay is a result of significant loss of resonance enhancement condition and electric polarizability, the conformational inhomogeneity of the chromophore still holds (i.e., some undergo ESPT to I*, while some undergo vibrational cooling within A*) [23,24,40]. An interesting correlation can be made to the previously observed vibrational cooling time constant of ~70 ps based on an adjacent 1265 cm^−1^ A* marker band for the SYG chromophore of a calcium biosensor GEX-GECO1 using Stokes FSRS with an 800 nm Raman pump [24]. We conjecture that this mainly C–O stretching motion could be more sensitive to the A* decay dynamics along the slope of the A* potential energy surface (PES), and once the ESPT proceeds from the lower portion of the A* state, a higher frequency mode (than the 1247 cm^−1^ mode, see Figure 6a and Figure S3b) appears, which can be analyzed to accurately retrieve the I* rise dynamics [4,11,21,23]. Moreover, the difference between this 82 ps time constant (1247 cm^−1^ mode decay, anti-Stokes FSRS with 504 nm Raman pump in Figure 6b) and 190 ps time constant (1583 cm^−1^ mode frequency blue shift, Stokes FSRS with 539 nm Raman pump in Figure 5c) stems from the mode-dependent nature of the trapped A* relaxation dynamics on a multidimensional excited state PES [12,52,55,64], as well as different dynamic resonance Raman conditions [31,52]. In particular, because the chromophore electronic absorption band typically red shifts upon gaining more charge transfer character in reaching a deprotonated state [32,68,69], the anti-Stokes FSRS resonance condition with a 504 nm Raman pump and a bluer Raman probe of ~455–500 nm highlights the more protonated A* species at early times, so the gradually diminishing A* SE band within ~200 ps (Figure 2) could contribute to a faster decay time constant of an A* Raman mode. By contrast, the Stokes FSRS with a 539 nm Raman pump and a redder Raman probe of ~545–605 nm, allows better tracking of more deprotonated A* species at later time points, with a more stagnant TA profile for the wavelength region above ~510 nm (Figure 2b).

On the photoproduct side, the 830 cm^−1^ mode reflects a relatively pure I* intensity rise (a negative peak becoming more negative) with a ~370 ps time constant. Although it has an initial small intensity, the early-time intensity is only ~13.5% of the overall intensity (Figure 6b). This could be reflective of an initial ESPT component along a pre-existing, largely optimal H-bonding chain, that leads to some I* population within ~1 ps (see Figure 2). While the anti-Stokes FSRS with a 504 nm Raman pump mainly enhances the A* species at early times, it also tracks the I* rise dynamics as the I* population accumulates at later times so overall there could be some interference or convolution effect. In comparison, the I* mode intensity rise, as reflected by the 622 cm^−1^ mode in Stokes FSRS with a 539 nm Raman pump, exhibits a larger initial intensity (~39% of the overall I* mode intensity, see above) and a smaller time constant of ~230 ps (Figure 5b), which indicates that the 539 nm Raman pump with redder Raman probe photons better enhance the deprotonated chromophore species, including those generated on the fs to few ps time scales [26,35] before the main ESPT step. In other words, due to a “cleaner” pre-resonance condition with the I* SE band only, the Stokes FSRS with a 539 nm Raman pump is a more reliable way to monitor the I* intensity rise on ultrafast time scales.

### 2.7. Different Raman Pumps Generate Characteristic Raman Line Shapes and Track Transient Species

In this comprehensive work, a systematic comparison between the transient Raman features along an ultrafast ESPT reaction inside a protein pocket is made possible by tuning the Raman pump to three strategic wavelength locations (801, 539, and 504 nm) from the near-IR to the visible region. The versatile selection of the Raman probe from a supercontinuum white light, across the near-UV to near-IR range, enables the FSRS to be readily collected on both the Stokes and anti-Stokes sides. A wealth of information becomes available as the photoreactant (A*) converts via an ESPT reaction into photoproduct (I*) species with intrinsic conformational inhomogeneity, so a portion of the A* species undergoes energy dissipation via other pathways (generally faster than the ESPT reaction inside the GFP-S205V mutant) as reflected by detailed analysis of the electronic and vibrational dynamics and associated time constants in a non-equilibrium state. During these dynamic processes, charge transfer likely occurs in concert with proton motions from the chromophore phenolic hydroxyl end to an internal water molecule and further down the H-bonding chain for ESPT (see Figure 1a). Depending upon the degree and stage of chromophore deprotonation, charge transfer mainly facilitates the dipole change for system–bath interactions as well as energy stabilization of the excited state prior to the main radiative pathway (i.e., fluorescence in this case). To further visualize the photochemical reaction inside GFP-S205V and highlight the sensitivity of tunable FSRS technique [8,10], Figure 7 directly contrasts the excited state Raman modes at two representative time delay points with three distinct Raman pump wavelengths. Besides characteristic line shapes, these vibrational mode frequencies clearly differ from their ground state counterparts, as shown in Figure 3, confirming their excited state nature and the rich information content we can glean by analyzing their temporal dynamics across the experimental time window (Figure 4, Figure 5 and Figure 6) and correlating with the fs-TA results (Figure 2).

At very early time (100 fs) in Figure 7a, the Raman modes belong to A* species, since they are the major population immediately following photoexcitation. With the tuning of the Raman pump–probe pair, most of the modes remain at similar positions, while some modes exhibit clear frequency shifts. For example, in FSRS with an 801 nm Raman pump, a peak doublet at 1139 and 1180 cm^−1^ is observed [4,24]. However, the 1180 cm^−1^ mode is prominent (as a positive peak, Raman gain) in FSRS with a 539 nm Raman pump, whereas the 1139 cm^−1^ mode is dominant (as a negative peak, Raman loss) in the anti-Stokes FSRS with a 504 nm Raman pump. We note that a much smaller negative peak at 1180 cm^−1^ also exists in the anti-Stokes FSRS spectrum. Since the 539 nm (pre-resonant with the I* SE band) and the 504 nm (pre-resonant with the A* SE band at early times) Raman pump focus on different species, this result indicates that the 1139 cm^−1^ mode is more related to the protonated chromophore, whereas the 1180 cm^−1^ mode has more contribution from a slightly deprotonated chromophore [13]. A related observation is the small peak intensity buildup at ~1170 cm^−1^ (i.e., higher frequency than the ~1139 cm^−1^ A* mode) past 100 ps in Figure 6a. Notably, complications could arise in the Stokes FSRS when the resonance conditions of the Raman pump become favorable to induce vibronic transitions involving certain Raman marker bands, and for the deprotonated chromophore of GFP-S205V, the ~1180 cm^−1^ mode merges into a broad dispersive line shape with the adjacent ~1240 cm^−1^ mode (see Figure 7a and Figure S3a for the raw experimental data with spectral baselines). For this reason, we cannot easily track its intensity dynamics as those reported for the related GFP derivatives [13,23,26].

At a much later time (600 ps) in Figure 7b, the Raman modes should be mostly coming from I* species, because the ESPT reaction has a main time constant of ~360 ps (Figure 2b). FSRS with an 800 nm Raman pump can hardly resolve any I* features [4,11] because the I* species has no ESA or SE band in this near-IR region. In FSRS with a 539 or 504 nm Raman pump, the I* Raman modes are clearly observed, and most of peaks appear at similar locations with an opposite sign corresponding to the Raman gain and loss in Stokes and anti-Stokes FSRS, respectively [10,31,52]. Moreover, the 1330 cm^−1^ mode displays a dispersive line shape in both FSRS measurements with a 539 or 504 nm Raman pump, confirming that this particular vibrational motion is strongly coupled with the electronic transitions from the I* state. Given the phase difference between the dispersive line shapes in this spectral region, the resonance conditions are different with the 539 nm Raman pump in Stokes FSRS (overlap with the I* SE band red shoulder) versus the 504 nm Raman pump in anti-Stokes FSRS (overlap with the I* SE band blue shoulder mixed with the I* ESA band red shoulder). Notably, the 1330 cm^−1^ mode consists of the chromophore bridge C–H rocking, phenolic ring-H rocking, and imidazolinone ring in-plane deformation (see Table 1), which is a very delocalized mode. As shown in Figure 5, Figure 6 and Figure 7, this particular mode rises at later times that correlate well with the ESPT time constant, and the mode dispersive line shape becomes more prominent as I* species accumulate (e.g., could be related to vibronic coupling). Therefore, this mode could effectively facilitate the I* species to interact with surrounding water molecules and protein residues to stabilize the nascent I* species on an ultrafast time scale, in accord with the high sensitivity of bridge-H motions to the protonation state change of the chromophore [4,13,21]. In essence, a significant involvement with improved electric polarizabilities of certain nuclear motions (not every mode, as shown in Figure 5 and Figure 6) along the CT and ESPT reaction coordinate on the excited state PES, pinpoints the effective dissipation pathway(s) of the photoexcitation energy via intra- and intermolecular interactions. Though at this point, we could not gain deeper insights due to the lack of knowledge about the exact time scale for intramolecular (e.g., within the chromophore) and intermolecular (e.g., between the chromophore and surrounding residues) motions in a protein pocket, it is plausible that primary structural motions on ultrafast time scales could have a notable effect on molecular events occurring on much longer timescales from large-scale proton transfer [4,12] and drug-enzyme binding [70] to other functions in chemical and biological systems [71].

**Figure 8:**
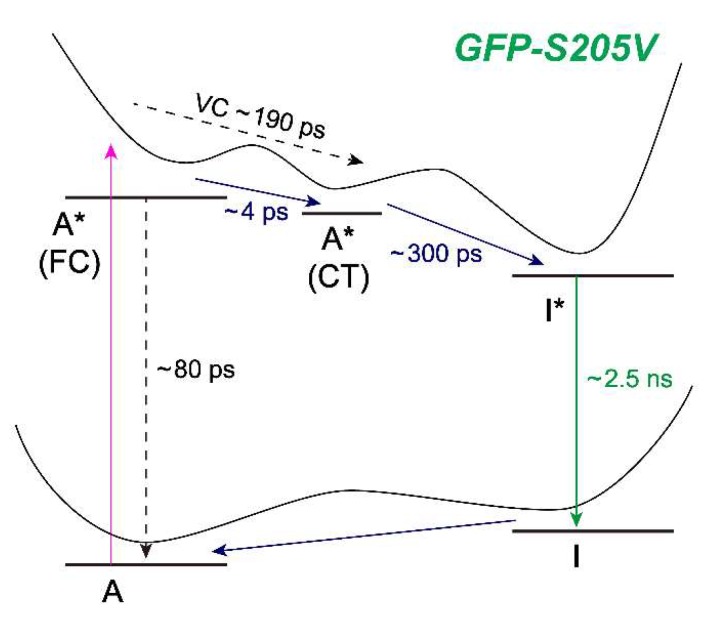
Photocycle kinetic scheme of the GFP-S205V mutant on the basis of fs-TA and excited state FSRS results. The pertinent protonated/deprotonated chromophore species in the electronic ground (S_0_) and excited state (S_1_) are depicted by horizontal bars with the associated state labels. The time constants uncovered in this work are denoted by the respective arrows between one state that evolves into another. The potential energy surfaces are illustrated with the transition barriers. In general, the dashed arrows represent the vibrational cooling (VC) pathways within the trapped A* species/subpopulations that do not undergo ESPT, and the 80 ps time constant could be attributed to a nonradiative pathway back to the ground state (A). See main text for a detailed discussion on the inhomogeneous distribution of A and A* populations in aqueous solution, and the proposed charge-transfer (CT) character of the intermediate A* species en route to the deprotonated chromophore in an unrelaxed protein environment (I*).

The other interesting observation is that the 622 cm^−1^ mode in FSRS with a 539 nm Raman pump splits into two modes (603 and 642 cm^−1^) in the anti-Stokes FSRS with a 504 nm Raman pump, indicative of conformational inhomogeneity of the nascent I* species, which are enhanced by a different electronic resonance condition achieved by the 504 nm Raman pump (because of its overlap with both the ESA and SE bands of I* at later times). Nevertheless, performing a series of tunable FSRS experiments with dynamic resonance conditions still provides an effective way to validate the excited state peak positions, as well as gain additional structural dynamics insights into the vibronic coupling, vibrational relaxation, and distinctive subpopulations of a transient species before and after a photochemical reaction (see Figure 8 for the photocycle kinetic scheme of GFP-S205V).

When comparing the raw experimental FSRS data with different Raman pumps (see Figure S3 in the Supplementary Materials), the conventional FSRS with an 800 nm Raman pump shows all positive Raman peaks and the flattest baseline due to a pre-resonance condition with a weak A* ESA band. Tunable FSRS with a 539 or 504 nm Raman pump, which achieves pre-resonance with the strong 513 nm I* SE band, the 478 nm A* SE band, or the ~445 nm I* ESA band (Figure 2b), all exhibit highly sloping baselines due to the Raman-pump-induced change to the TA profile [12,72], and the resultant dispersive line shapes of certain excited state Raman modes [52]. The drawing of smooth and largely featureless spectral baselines (see Figure S3 for example) allows subtraction of the TA-like electronic background, so the much narrower vibrational features (i.e., Raman modes) can be obtained with high fidelity [8,10,12]. Notably, the low-frequency modes and the characteristic I* modes can only be clearly resolved in the tunable FSRS with a blue Raman pump. Consequently, a combination of various Raman pumps can be used to cross-examine the excited state Raman bands throughout a photoinduced process on crucial molecular time scales, to gain a more complete understanding of a complicated system like the GFP-S205V mutant in aqueous solution.

## 3. Materials and Methods

### 3.1. Protein Sample Preparation and Steady-State Spectroscopic Characterization

The site-specific mutagenesis and protein expression were described in an earlier publication [38]. The electronic absorption and emission spectra of the protein sample in aqueous buffer solution (pH = 7.9) were collected by a Thermo Scientific Evolution 201 UV–visible (UV–vis, Waltham, MA, USA) and a Hitachi F-2500 fluorescence spectrophotometer (Chatsworth, CA, USA), respectively. Given that the p*K*_a_ of the chromophore inside GFP-S205V was estimated to be over 11, due to the increased hydrophobicity of the protein pocket and the trend from titration analysis of the A and B absorption peaks at ~395 and 490 nm (Figure 1b) [38,61], the chromophore remains predominantly in the protonated state at thermal equilibrium at room temperature (i.e., the A absorption peak dominates the ground state absorption spectrum).

### 3.2. Femtosecond Transient Absorption and Stimulated Raman Optical Setups

The time-resolved experiments were based on a femtosecond (fs) laser system consisting of a mode-locked Ti:sapphire oscillator (Mantis-5, Coherent) and regenerative amplifier (Legend Elite-USP-1K-HE, Coherent, Inc., Santa Clara, CA, USA) with a fundamental output with ∼35 fs pulse duration, ∼800 nm center wavelength, 4 mJ pulse energy at 1 kHz repetition rate. In both the fs transient absorption (fs-TA) and FSRS experiments, the actinic pump was centered at 400 nm, generated by frequency doubling part of the laser output using a BBO crystal (Type I, cutting angle 27.8°, 0.3 mm thickness). The polarization of this photoexcitation pulse was rotated 90° by a λ/2 waveplate, and then compressed by a UV-grade fused silica prism pair (06SB10, Newport, Inc., Irvine, CA, USA) to ~40 fs. To manage the time interval after excitation, the actinic pump is delayed by a computer-controlled stepper-motor-driven linear translation stage. To generate a proper probe pulse, a small portion of the fundamental laser output was focused on a *Z*-cut sapphire plate (2 or 3 mm thick) to generate the supercontinuum white light spanning from the near UV to near IR [11]. For the fs-TA experiment, with the results shown in Figure 2, the visible part of the white light is selected and temporally compressed by a chirped mirror pair (DCM-12, 400–700 nm, Laser Quantum, Inc., Santa Clara, CA, USA) to ~40 fs pulse duration, before focusing on the same sample spot with the 400 nm pump pulse (~0.1 µJ pulse energy). The cross-correlation time between the compressed pump and probe pulses was measured to be ~60 fs in a 1 mm pathlength quartz cuvette (1-Q-1, Starna Cells, Inc., Atascadero, CA, USA) filled with standard solvent such as methanol or water.

In conventional FSRS setup with experimental spectra shown in Figure 4, the picosecond (ps) 800 nm Raman pump pulse (∼10 cm^−1^ bandwidth) was produced by a home-built grating-based spectral filter, which includes a gold reflective grating (1200 grooves/mm, 750 nm blaze) and a cylindrical lens [29,46]. The Stokes Raman probe was the near-IR part of the aforementioned supercontinuum white light (in a 2 mm-thick sapphire plate), which was selected using a long-pass filter (color glass filter RG830, Newport, Inc., Irvine, CA, USA) and compressed in the time domain by a fused silica prism pair (AFS-FS, Thorlabs, Inc., Newton, NJ, USA) to ~35 fs pulse duration.

In the tunable FSRS setup with 539 nm (results in Figure 5) and 504 nm (results in Figure 6) Raman pump, the detailed experimental procedures were previously reported [11,73,74,75]. In brief, the generation of narrowband Raman pump in the visible range (from ca. 480–750 nm, up to 14 µJ/pulse) consists of three major home-built parts: a second harmonic bandwidth compressor (SHBC) to generate a ps 400 nm pump; a femtosecond noncollinear optical parametric amplifier (NOPA) in conjunction with a grating-slit-based spectral filter to produce a ps seed; and a two-stage picosecond NOPA to amplify the ps seed with the ps 400 nm pump. To work with this visible Raman pump as a pair to stimulate Raman signals in either the Stokes or anti-Stokes regime, the corresponding Raman probe pulse was selected from the above-mentioned supercontinuum white light based on laser focusing on a 3 mm-thick sapphire plate, followed by temporal compression via a chirped mirror pair (DCM-9, 450–950 nm, Laser Quantum, Inc., Santa Clara, CA, USA).

In the FSRS sample measurement scheme, the actinic pump, Raman pump, and probe beams were focused onto the solution sample housed in a 1 mm-thick quartz cell (1-Q-1, see above) by a parabolic reflective mirror (*RFL* = 101.6 mm, MPD249-F01, Thorlabs, Inc., Newton, NJ, USA). A similar 400 nm, fs photoexcitation pulse energy of ~300 nJ was used in all three FSRS measurements, while for the ps Raman pump, ~1 µJ/pulse was used in tunable FSRS (i.e., 539 and 504 nm Raman pump) and ~2 µJ/pulse was used in conventional FSRS (800 nm Raman pump). Past the sample, the transmitted actinic pump and Raman pump beams were spatially blocked by an iris, while the Raman probe beam was collimated and focused into an imaging spectrograph (Acton SpectraPro SP-2356, 300 mm focal length, Princeton Instruments, Inc., Trenton, NJ, USA) with a 1200 (600) grooves/mm, 500 (1000) nm blaze grating in tunable (conventional) FSRS, and then imaged on a CCD array camera (PIXIS:100F, Princeton Instruments, Inc., Trenton, NJ, USA) synchronized with the fundamental laser repetition clock for spectral data collection by a computer LabVIEW suite. To better capture the early-time primary structural events that may involve quantum beats/spectral oscillations [4,9,12], we collected the excited state FSRS data with 50 fs step size from negative time points to 2 ps, and 1 ps step size from 2–10 ps (see Figure S2 for a linear plot on the relevant time axis, as well as a cluster of spectral data points around 1 ps in a semilogarithmic plot like Figure 4b). During the excited state, FSRS data scan (with 3000 laser shots hence 1500 Raman spectra per time point, and repeated for 8 times, so 12,000 spectra were averaged for each excited state spectrum displayed in Figure 4, Figure 5, Figure 6 and Figure 7), the protein samples were constantly stirred by a miniature magnetic staple wrapped by a thin layer of parafilm, and the UV–vis spectra were collected before and after each FSRS measurement to confirm sample integrity with a typical electronic spectral change of <5%. The protein sample concentration for TA (FSRS) experiments was made to reach OD ≈ 0.4 (1.0) per mm at 400 nm.

### 3.3. DFT and TD-DFT Calculations

To understand the nature of vibrational normal modes of the SYG chromophore, quantum chemical calculations of the neutral (protonated) and anionic (deprotonated) chromophore were performed in Gaussian 09 software [60]. For the ground state (S_0_) Raman mode frequencies, we used the density functional theory (DFT) calculation of an optimized protonated or deprotonated SYG chromophore (capped with methyl groups) at the RB3LYP level with 6-31G+(d,p) basis sets in vacuo (mimicking the overall hydrophobic environment inside a protein pocket). To obtain the vibrational frequencies of a geometrically optimized, protonated, or deprotonated SYG chromophore in the excited state (S_1_ in this case), a time-dependent DFT(TD-DFT) calculation with the same theory level and basis sets was conducted. The output normal mode frequencies were multiplied by a scaling factor of 0.97 and 0.96 to compare with the experimental peak values in S_0_ and S_1_, respectively.

## 4. Conclusions

In summary, we implemented the fs-TA experiment and the wavelength-tunable FSRS with three strategic Raman pump wavelengths (i.e., 800, 539, and 504 nm) to systematically dissect the excited state structural evolution of a GFP single-site mutant S205V, which has the same SYG chromophore as wtGFP but a different local H-bonding network and significantly prolonged ESPT reaction. The dynamic changes occurring to the vibrational properties of an organic molecule are monitored when processes of proton transfer are suitably excited by photons inside a biomolecule. Combining results from ultrafast electronic and vibrational spectroscopies, the ESPT time constant is retrieved to be ~300 ps. The 800 nm Raman pump allows the effective monitoring of key vibrational dynamics of the photoreactant A* species. The 539 nm Raman pump focuses on the nascent I* features at later times, but also sheds light on a slightly different A* species with a similar ESPT time constant. This Raman pump uncovers a vibrational cooling process of the trapped A* species on the ~190 ps time scale, faster than the ESPT reaction that occurs in parallel. Performing the anti-Stokes FSRS with a 504 nm Raman pump further resolves an A* species with more protonated character, which could undergo a faster vibrational relaxation or radiationless transition with a ~80 ps time constant. A ~1330 cm^−1^ I* mode is considered to be strongly coupled to the I* electronic transitions to nearby states (e.g., S_0_, S_2_) due to the observation of clear dispersive line shapes in multiple FSRS datasets from the Stokes to the anti-Stokes regime. Based on the chromophore vibrational mode assignment from quantum calculations, this delocalized mode with significant bridge-H motions between the two rings could facilitate the stabilization of the nascent I* species into the final fluorescent state. We suggest that an atomic modification of the bridge region, including the change of the bridge H to other bulky or electron withdrawing groups, could effectively change the fluorescence outcome, including emission color and quantum yield, which on the characterization front, should display distinct Raman peak line shapes and dynamics, as shown in Figure 5, Figure 6 and Figure 7. During the molecular engineering process, the species-dependent vibrational information from tunable FSRS could then potentially validate or refute certain reaction routes, leading to target functionality such as brighter fluorescence, higher photostability or less photobleaching.

These spectral results reveal that, for an intrinsically inhomogeneous system like an embedded chromophore inside a protein pocket, multiple Raman pump wavelengths tuned across the broad spectral region with various transient electronic features are preferred and advantageous to track a photochemical reaction between different molecular species. Selective and dynamic resonance enhancement opens the door to unravel a series of concomitant pathways with different vibrational probes, and sheds light on the functionally relevant multidimensional potential energy landscape of the molecular system from the ground to excited state (see Figure 8 for illustration). The exhaustive insights into the conformational dynamics of the protein chromophore by ultrafast electronic and vibrational spectroscopies allow us to estimate the energy levels and characteristic lifetimes of the excited states related to proton transfer processes. Along the multidimensional excited state PES, elucidation of characteristic/active nuclear motions holds the key to understanding photochemical reaction mechanisms. Furthermore, this work shows, for the first time, that Stokes and anti-Stokes FSRS can be applied to study the photoinduced proton transfer reaction in proteins, with new knowledge about how the inhomogeneous distribution of photoreactant species converts to the photoproduct species prior to fluorescence. Our findings provide useful guidelines for such real-time atomic tracking in functional photosensitive systems, and a vivid illustration with a fundamental level of details regarding a photochemical reaction, in particular excited state proton transfer, which powers the fluorescence of GFP and its derivatives in numerous bioimaging applications.

## Figures and Tables

**Table 1:** Vibrational assignment for the I* excited state Raman bands of the GFP-S205V mutant.

Exp. Freq. (cm^−1^) ^a^	Cal. Freq. (cm^−1^) ^b^	Vibrational Mode Assignment (Major Atomic Motions)
454	478	imidazolinone ring and phenol ring out-of-plane (OOP) deformation
603/622/642	619	imidazolinone ring and phenol ring in-plane deformation
726	729	imidazolinone ring OOP deformation with small-scale phenol ring OOP deformation
826/830	805	phenol ring breathing, bridge CCC bending with small-scale imidazolinone ring deformation
1295/1324/1340	1348	bridge C–H rocking, phenolic ring-H rocking, and imidazolinone ring in-plane deformation

^a^ The observed excited state (S_1_) Raman mode frequencies of the deprotonated SYG chromophore from Stokes FSRS with a 539 nm Raman pump (Figure 5a) and anti-Stokes FSRS with a 504 nm Raman pump (Figure 6a). The closeness of the mode frequencies in these independent experiments with different optical setups on the same protein sample in aqueous buffer solution confirms the nature of the same vibrational motion. ^b^ Excited state vibrational frequencies of a geometrically optimized deprotonated SYG chromophore in the singlet excited state (S_1_) are calculated in Gaussian 09 [60] using the time-dependent density functional theory (TD-DFT) RB3LYP 6-31G+(d,p) in vacuo. The calculated normal mode frequencies are scaled with a factor of 0.96.

## Supplementary Materials

The following information is available online, Figure S1: Global analysis of the fs-TA spectra with a sequential kinetic model. Figure S2: Excited state intensity dynamics of the 1180 cm^−1^ mode of the GFP-S205V chromophore in conventional FSRS including negative time points. Figure S3: Raw experimental excited state FSRS data at two representative time points with spectral baselines.

## References

[1] Hochstrasser R.M. (2007). Two-dimensional spectroscopy at infrared and optical frequencies. Proc. Natl. Acad. Sci. USA.

[2] Błasiak B., Londergan C.H., Webb L.J., Cho M. (2017). Vibrational probes: From small molecule solvatochromism theory and experiments to applications in complex systems. Acc. Chem. Res..

[3] Kukura P., McCamant D.W., Yoon S., Wandschneider D.B., Mathies R.A. (2005). Structural observation of the primary isomerization in vision with femtosecond-stimulated Raman. Science.

[4] Fang C., Frontiera R.R., Tran R., Mathies R.A. (2009). Mapping GFP structure evolution during proton transfer with femtosecond Raman spectroscopy. Nature.

[5] Yoshizawa M., Kurosawa M. (1999). Femtosecond time-resolved Raman spectroscopy using stimulated Raman scattering. Phys. Rev. A.

[6] McCamant D.W., Kukura P., Yoon S., Mathies R.A. (2004). Femtosecond broadband stimulated Raman spectroscopy: Apparatus and methods. Rev. Sci. Instrum..

[7] Kukura P., McCamant D.W., Mathies R.A. (2007). Femtosecond stimulated Raman spectroscopy. Annu. Rev. Phys. Chem..

[8] Dietze D.R., Mathies R.A. (2016). Femtosecond stimulated Raman spectroscopy. ChemPhysChem.

[9] Hoffman D.P., Mathies R.A. (2016). Femtosecond stimulated Raman exposes the role of vibrational coherence in condensed-phase photoreactivity. Acc. Chem. Res..

[10] Fang C., Tang L., Oscar B.G., Chen C. (2018). Capturing structural snapshots during photochemical reactions with ultrafast Raman spectroscopy: From materials transformation to biosensor responses. J. Phys. Chem. Lett..

[11] Tang L., Liu W., Wang Y., Zhao Y., Oscar B.G., Campbell R.E., Fang C. (2015). Unraveling ultrafast photoinduced proton transfer dynamics in a fluorescent protein biosensor for Ca^2+^ imaging. Chem. Eur. J..

[12] Liu W., Wang Y., Tang L., Oscar B.G., Zhu L., Fang C. (2016). Panoramic portrait of primary molecular events preceding excited state proton transfer in water. Chem. Sci..

[13] Tang L., Liu W., Wang Y., Zhu L., Han F., Fang C. (2016). Ultrafast structural evolution and chromophore inhomogeneity inside a green-fluorescent-protein-based Ca^2+^ biosensor. J. Phys. Chem. Lett..

[14] Shim S., Dasgupta J., Mathies R.A. (2009). Femtosecond time-resolved stimulated Raman reveals the birth of bacteriorhodopsin’s J and K intermediates. J. Am. Chem. Soc..

[15] Polli D., Altoè P., Weingart O., Spillane K.M., Manzoni C., Brida D., Tomasello G., Orlandi G., Kukura P., Mathies R.A. (2010). Conical intersection dynamics of the primary photoisomerization event in vision. Nature.

[16] Dasgupta J., Frontiera R.R., Taylor K.C., Lagarias J.C., Mathies R.A. (2009). Ultrafast excited-state isomerization in phytochrome revealed by femtosecond stimulated Raman spectroscopy. Proc. Natl. Acad. Sci. USA.

[17] Ferrante C., Pontecorvo E., Cerullo G., Vos M.H., Scopigno T. (2016). Direct observation of subpicosecond vibrational dynamics in photoexcited myoglobin. Nat. Chem..

[18] Weigel A., Dobryakov A., Klaumünzer B., Sajadi M., Saalfrank P., Ernsting N.P. (2011). Femtosecond stimulated Raman spectroscopy of flavin after optical excitation. J. Phys. Chem. B.

[19] Hall C.R., Heisler I.A., Jones G.A., Frost J.E., Gil A.A., Tonge P.J., Meech S.R. (2017). Femtosecond stimulated Raman study of the photoactive flavoprotein AppABLUF. Chem. Phys. Lett..

[20] Nakamura R., Hamada N., Abe K., Yoshizawa M. (2012). Ultrafast hydrogen-bonding dynamics in the electronic excited state of photoactive yellow protein revealed by femtosecond stimulated Raman spectroscopy. J. Phys. Chem. B.

[21] Oscar B.G., Liu W., Zhao Y., Tang L., Wang Y., Campbell R.E., Fang C. (2014). Excited-state structural dynamics of a dual-emission calmodulin-green fluorescent protein sensor for calcium ion imaging. Proc. Natl. Acad. Sci. USA.

[22] Wang Y., Tang L., Liu W., Zhao Y., Oscar B.G., Campbell R.E., Fang C. (2015). Excited state structural events of a dual-emission fluorescent protein biosensor for Ca^2+^ imaging studied by femtosecond stimulated Raman spectroscopy. J. Phys. Chem. B.

[23] Tachibana S.R., Tang L., Wang Y., Zhu L., Liu W., Fang C. (2017). Tuning calcium biosensors with a single-site mutation: Structural dynamics insights from femtosecond Raman spectroscopy. Phys. Chem. Chem. Phys..

[24] Tang L., Wang Y., Liu W., Zhao Y., Campbell R.E., Fang C. (2017). Illuminating photochemistry of an excitation ratiometric fluorescent protein calcium biosensor. J. Phys. Chem. B.

[25] Kuramochi H., Takeuchi S., Yonezawa K., Kamikubo H., Kataoka M., Tahara T. (2017). Probing the early stages of photoreception in photoactive yellow protein with ultrafast time-domain Raman spectroscopy. Nat. Chem..

[26] Tang L., Wang Y., Zhu L., Kallio K., Remington S.J., Fang C. (2018). Photoinduced proton transfer inside an engineered green fluorescent protein: A stepwise-concerted-hybrid reaction. Phys. Chem. Chem. Phys..

[27] Hall C.R., Conyard J., Heisler I.A., Jones G., Frost J., Browne W.R., Feringa B.L., Meech S.R. (2017). Ultrafast dynamics in light-driven molecular rotary motors probed by femtosecond stimulated Raman spectroscopy. J. Am. Chem. Soc..

[28] Hall C.R., Browne W.R., Feringa B.L., Meech S.R. (2018). Mapping the excited-state potential energy surface of a photomolecular motor. Angew. Chem. Int. Ed..

[29] Liu W., Han F., Smith C., Fang C. (2012). Ultrafast conformational dynamics of pyranine during excited state proton transfer in aqueous solution revealed by femtosecond stimulated Raman spectroscopy. J. Phys. Chem. B.

[30] Wang Y., Liu W., Tang L., Oscar B., Han F., Fang C. (2013). Early time excited-state structural evolution of pyranine in methanol revealed by femtosecond stimulated Raman spectroscopy. J. Phys. Chem. A.

[31] Liu W., Tang L., Oscar B.G., Wang Y., Chen C., Fang C. (2017). Tracking ultrafast vibrational cooling during excited-state proton transfer reaction with anti-Stokes and Stokes femtosecond stimulated Raman spectroscopy. J. Phys. Chem. Lett..

[32] Chen C., Liu W., Baranov M.S., Baleeva N.S., Yampolsky I.V., Zhu L., Wang Y., Shamir A., Solntsev K.M., Fang C. (2017). Unveiling structural motions of a highly fluorescent superphotoacid by locking and fluorinating the GFP chromophore in solution. J. Phys. Chem. Lett..

[33] Tsien R.Y. (1998). The green fluorescent protein. Annu. Rev. Biochem..

[34] Sanders J.K.M., Jackson S.E. (2009). The discovery and development of the green fluorescent protein, GFP. Chem. Soc. Rev..

[35] Chattoraj M., King B.A., Bublitz G.U., Boxer S.G. (1996). Ultra-fast excited state dynamics in green fluorescent protein: Multiple states and proton transfer. Proc. Natl. Acad. Sci. USA.

[36] Ormö M., Cubitt A.B., Kallio K., Gross L.A., Tsien R.Y., Remington S.J. (1996). Crystal structure of the *aequorea victoria* green fluorescent protein. Science.

[37] Kennis J.T.M., Larsen D.S., van Stokkum I.H.M., Vengris M., van Thor J.J., van Grondelle R. (2004). Uncovering the hidden ground state of green fluorescent protein. Proc. Natl. Acad. Sci. USA.

[38] Shu X., Leiderman P., Gepshtein R., Smith N.R., Kallio K., Huppert D., Remington S.J. (2007). An alternative excited-state proton transfer pathway in green fluorescent protein variant S205V. Protein Sci..

[39] Simkovitch R., Huppert A., Huppert D., Remington S.J., Miller Y. (2013). Proton transfer in wild-type GFP and S205V mutant is reduced by conformational changes of residues in the proton wire. J. Phys. Chem. B.

[40] Laptenok S.P., Lukacs A., Gil A., Brust R., Sazanovich I.V., Greetham G.M., Tonge P.J., Meech S.R. (2015). Complete proton transfer cycle in GFP and its T203V and S205V mutants. Angew. Chem. Int. Ed..

[41] Salna B., Benabbas A., Sage J.T., van Thor J., Champion P.M. (2016). Wide-dynamic-range kinetic investigations of deep proton tunnelling in proteins. Nat. Chem..

[42] Creemers T.M.H., Lock A.J., Subramaniam V., Jovin T.M., Völker S. (1999). Three photoconvertible forms of green fluorescent protein identified by spectral hole-burning. Nat. Struct. Biol..

[43] Berera R., van Grondelle R., Kennis J.T.M. (2009). Ultrafast transient absorption spectroscopy: Principles and application to photosynthetic systems. Photosynth. Res..

[44] Snellenburg J.J., Laptenok S., Seger R., Mullen K.M., van Stokkum I.H.M. (2012). Glotaran: A Java-based graphical user interface for the R package TIMP. J. Stat. Softw..

[45] Rini M., Magnes B.-Z., Pines E., Nibbering E.T.J. (2003). Real-time observation of bimodal proton transfer in acid-base pairs in water. Science.

[46] Han F., Liu W., Fang C. (2013). Excited-state proton transfer of photoexcited pyranine in water observed by femtosecond stimulated Raman spectroscopy. Chem. Phys..

[47] Vendrell O., Gelabert R., Moreno M., Lluch J.M. (2006). Potential energy landscape of the photoinduced multiple proton-transfer process in the green fluorescent protein:  Classical molecular dynamics and multiconfigurational electronic structure calculations. J. Am. Chem. Soc..

[48] Batignani G., Pontecorvo E., Giovannetti G., Ferrante C., Fumero G., Scopigno T. (2016). Electronic resonances in broadband stimulated Raman spectroscopy. Sci. Rep..

[49] Xu J., Plaxco K.W., Allen S.J. (2009). Probing the collective vibrational dynamics of a protein in liquid water by terahertz absorption spectroscopy. Protein Sci..

[50] Hamm P., Meuwly M., Johnson S.L., Beaud P., Staub U. (2017). Perspective: THz-driven nuclear dynamics from solids to molecules. Struct. Dyn..

[51] Umapathy S., Lakshmanna A., Mallick B. (2009). Ultrafast Raman loss spectroscopy. J. Raman Spectrosc..

[52] Oscar B.G., Chen C., Liu W., Zhu L., Fang C. (2017). Dynamic Raman line shapes on an evolving excited-state landscape: Insights from tunable femtosecond stimulated Raman spectroscopy. J. Phys. Chem. A.

[53] Bragg A.E., Yu W., Zhou J., Magnanelli T. (2016). Ultrafast Raman spectroscopy as a probe of local structure and dynamics in photoexcited conjugated materials. J. Phys. Chem. Lett..

[54] Quick M., Dobryakov A.L., Kovalenko S.A., Ernsting N.P. (2015). Resonance femtosecond-stimulated Raman spectroscopy without actinic excitation showing low-frequency vibrational activity in the S_2_ state of all-trans β-carotene. J. Phys. Chem. Lett..

[55] Roy K., Kayal S., Ariese F., Beeby A., Umapathy S. (2017). Mode specific excited state dynamics study of bis(phenylethynyl)benzene from ultrafast Raman loss spectroscopy. J. Chem. Phys..

[56] Bell A.F., He X., Wachter R.M., Tonge P.J. (2000). Probing the ground state structure of the green fluorescent protein chromophore using Raman spectroscopy. Biochemistry.

[57] Yang F., Moss L.G., Phillips G.N. (1996). The molecular structure of green fluorescent protein. Nat. Biotechnol..

[58] Brejc K., Sixma T.K., Kitts P.A., Kain S.R., Tsien R.Y., Ormö M., Remington S.J. (1997). Structural basis for dual excitation and photoisomerization of the *Aequorea victoria* green fluorescent protein. Proc. Natl. Acad. Sci. USA.

[59] Shinobu A., Palm G.J., Schierbeek A.J., Agmon N. (2010). Visualizing proton antenna in a high-resolution green fluorescent protein structure. J. Am. Chem. Soc..

[60] Frisch M.J., Trucks G.W., Schlegel H.B., Scuseria G.E., Robb M.A., Cheeseman J.R., Scalmani G., Barone V., Mennucci B., Petersson G.A. (2009). Gaussian 09, revision B.1.

[61] Shu X. (2007). Photophysics of Emission Color in Fluorescence Proteins. Ph.D. Thesis.

[62] Simkovitch R., Karton-Lifshin N., Shomer S., Shabat D., Huppert D. (2013). Ultrafast excited-state proton transfer to the solvent occurs on a hundred-femtosecond time-scale. J. Phys. Chem. A.

[63] Hamm P., Ohline S.M., Zinth W. (1997). Vibrational cooling after ultrafast photoisomerization of azobenzene measured by femtosecond infrared spectroscopy. J. Chem. Phys..

[64] Tang L., Wang Y., Zhu L., Lee C., Fang C. (2018). Correlated molecular structural motions for photoprotection after deep-UV irradiation. J. Phys. Chem. Lett..

[65] Lian T., Locke B., Kholodenko Y., Hochstrasser R.M. (1994). Energy flow from solute to solvent probed by femtosecond IR spectroscopy: Malachite green and heme protein solutions. J. Phys. Chem..

[66] Chen C., Zhu L., Fang C. (2018). Femtosecond stimulated Raman line shapes: Dependence on resonance conditions of pump and probe pulses. Chin. J. Chem. Phys..

[67] McHale J.L. (1999). Molecular Spectroscopy.

[68] Tominaga K., Walker G.C., Jarzeba W., Barbara P.F. (1991). Ultrafast charge separation in ADMA: Experiment, simulation, and theoretical issues. J. Phys. Chem..

[69] Vengris M., van Stokkum I.H.M., He X., Bell A.F., Tonge P.J., van Grondelle R., Larsen D.S. (2004). Ultrafast excited and ground-state dynamics of the green fluorescent protein chromophore in solution. J. Phys. Chem. A.

[70] Fang C., Bauman J.D., Das K., Remorino A., Arnold E., Hochstrasser R.M. (2008). Two-dimensional infrared spectra reveal relaxation of the nonnucleoside inhibitor TMC278 complexed with the HIV-1 reverse transcriptase. Proc. Natl. Acad. Sci. USA.

[71] Scholes G.D., Fleming G.R., Chen L.X., Aspuru-Guzik A., Buchleitner A., Coker D.F., Engel G.S., van Grondelle R., Ishizaki A., Jonas D.M. (2017). Using coherence to enhance function in chemical and biophysical systems. Nature.

[72] Lee J., Challa J.R., McCamant D.W. (2013). Pump power dependence in resonance femtosecond stimulated Raman spectroscopy. J. Raman Spectrosc..

[73] Zhu L., Liu W., Fang C. (2014). A versatile femtosecond stimulated Raman spectroscopy setup with tunable pulses in the visible to near infrared. Appl. Phys. Lett..

[74] Zhu L., Liu W., Wang Y., Fang C. (2015). Sum-frequency-generation-based laser sidebands for tunable femtosecond Raman spectroscopy in the ultraviolet. Appl. Sci..

[75] Tang L., Zhu L., Wang Y., Fang C. (2018). Uncovering the hidden excited state toward fluorescence of an intracellular pH indicator. J. Phys. Chem. Lett..

