# Supplementary material for: Excited State Structural Evolution of a GFP Single-Site Mutant Tracked by Tunable Femtosecond-Stimulated Raman Spectroscopy
Source: Molecules. 2018 Sep 1;23(9):2226. doi: 10.3390/molecules23092226 (PMC6225354; doi:10.3390/molecules23092226)
Supplement: Supplementary file 1 [file molecules-23-02226-s001.pdf]

Supplementary Materials (SM) for:

Excited State Structural Evolution of a GFP Single-Site Mutant Tracked by Tunable Femtosecond Stimulated Raman Spectroscopy

Longteng Tang [1] , Liangdong Zhu [1] , Miles A. Taylor [1] , Yanli Wang [1] , S. James Remington [2] and Chong Fang [1,]*

[1]Department of Chemistry, Oregon State University, Corvallis, Oregon 97331, USA

[2]Institute of Molecular Biology and Department of Physics, University of Oregon, Eugene, Oregon 97403, USA

Corresponding Author

*E-mail: Chong.Fang@oregonstate.edu. Phone:541-737-6704.

*This PDF file includes **SM Figures** S1-S3 on page S2-S4 and **SM References** on page S5.*

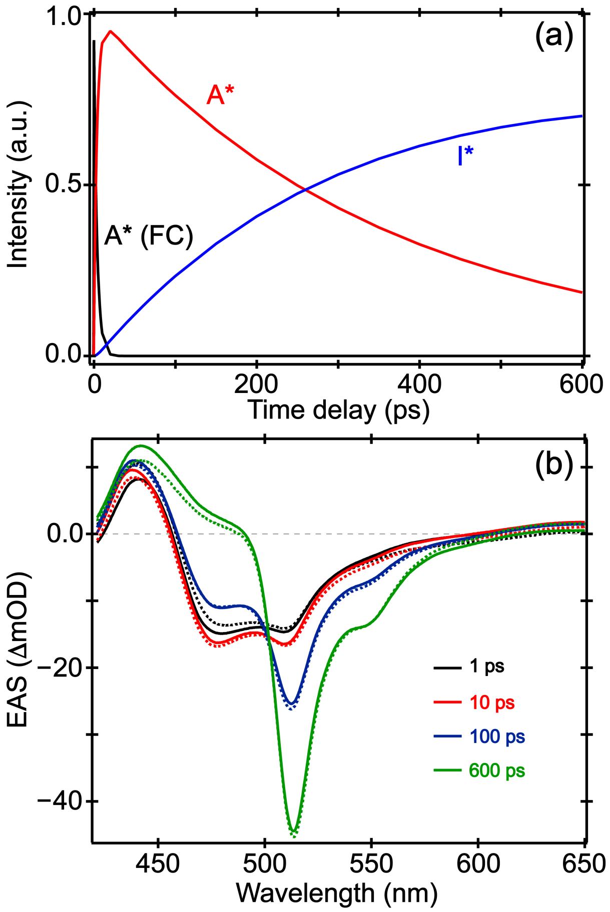

Figure S1. Global analysis of the fs-TA spectra of GFP-S205V after 400 nm photoexcitation. (a) Plot of the dynamics of three components from a sequential kinetic model, which consists of the A* (Franck-Condon region or FC, black) → A* (likely with charge-transfer character, red) [1,2] → I* (deprotonated chromophore in an unrelaxed environment, blue) speciation in the electronic excited state. The corresponding evolution-associated spectra (EAS) are displayed in Figure 2b (see main text). (b) Kinetic data traces (dotted curves) are overlaid with the global fits (solid curves) using Glotaran software [3] at representative time delay points of 1 ps (black), 10 ps (red), 100 ps (blue), and 600 ps (green) after electronic excitation. The time-dependent fitting traces largely match the experimental traces across the detection wavelength region, indicating that the kinetic model well approximates the fs-TA data from the ps to hundreds of ps time scale.

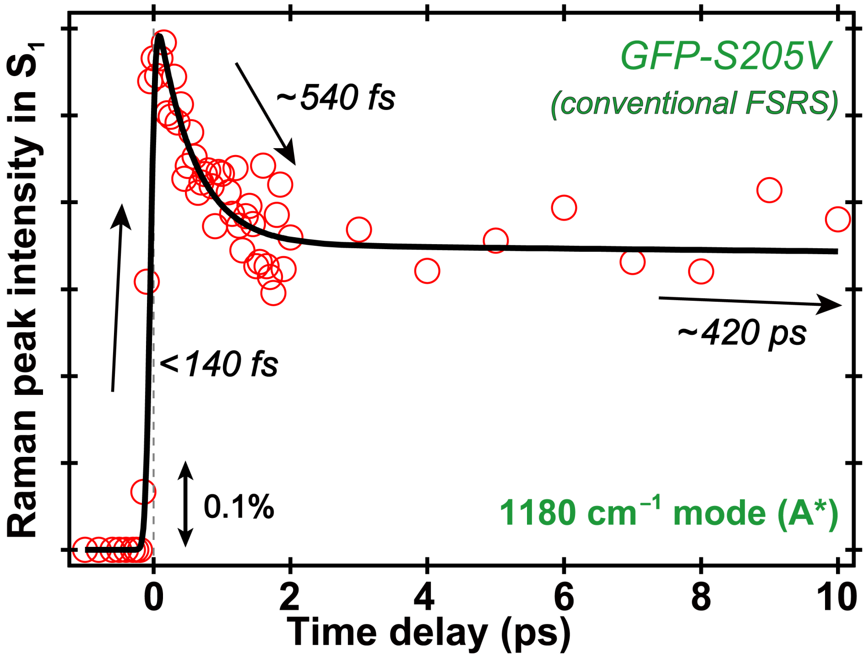

Figure S2. Excited state intensity dynamics of the 1180 cm^{-1} mode of the GFP-S205V protein chromophore upon 400 nm photoexcitation (including negative time points) using conventional FSRS, wherein the Raman pump is at 801 nm. The time window from –1 ps to 10 ps is shown, and the excited state Raman mode intensity data points (red circles) are overlaid with the least-squares multi-exponential fits (black curve). The time constant of the signal rise component near time zero (gray dashed line) is smaller than the cross-correlation time of the setup (140 fs, see Section 2.4 in main text). The semilogarithmic plot for a time window up to 600 ps can be seen in Figure 4b (main text), which better shows the long decay time constant of 420 ps.

Notably, the lack of clear spectral oscillations (quantum beats) within the initial ~2 ps differs from the wild-type GFP [4], which could be due to the dramatically altered ESPT chain inside the GFP-S205V mutant [5]. Besides suitable resonance conditions, the fs Raman observation of coherent mode intensity or frequency oscillations depends on the intricate interplay between the photoexcited chromophore and its local environment [6,7].

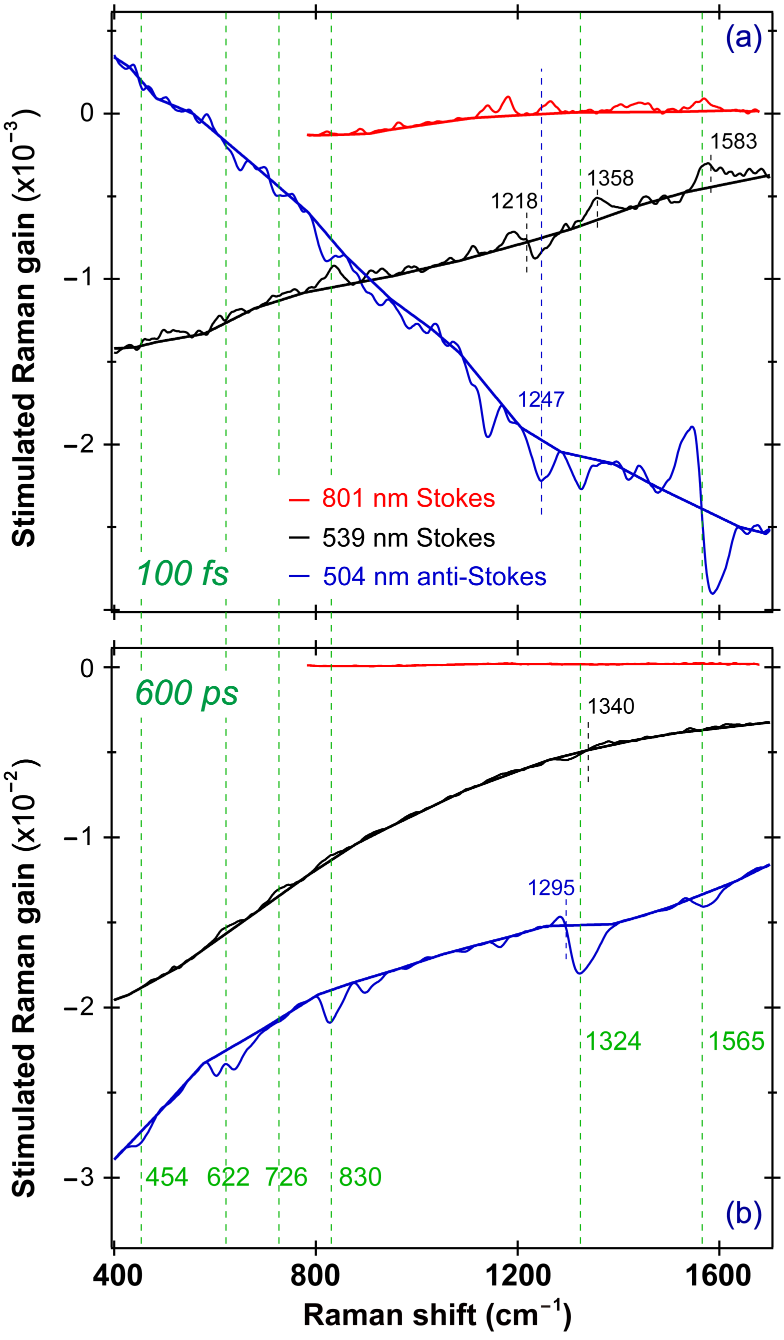

Figure S3. Raw experimental excited state FSRS spectra of GFP-S205V with Raman pump at 801 nm (red) in Stokes FSRS, 539 nm (black) in Stokes FSRS, and 504 nm (blue) in anti-Stokes FSRS at (a) 100 fs and (b) 600 ps time delay after 400 nm photoexcitation. The spline baselines are shown in color-coded solid curves. The vertical green, blue, and black dashed lines highlight the Raman marker bands shown in Figures 4, 5, 6, and 7 (main text).

SM References

1. Chen, C.; Liu, W.; Baranov, M.S.; Baleeva, N.S.; Yampolsky, I.V.; Zhu, L.; Wang, Y.; Shamir, A.; Solntsev, K.M.; Fang, C. Unveiling structural motions of a highly fluorescent superphotoacid by locking and fluorinating the GFP chromophore in solution. *J. Phys. Chem. Lett.* **2017**, *8*, 5921-5928.
2. Tang, L.; Wang, Y.; Zhu, L.; Kallio, K.; Remington, S.J.; Fang, C. Photoinduced proton transfer inside an engineered green fluorescent protein: A stepwise-concerted-hybrid reaction. *Phys. Chem. Chem. Phys.* **2018**, *20*, 12517-12526.
3. Snellenburg, J.J.; Laptenok, S.; Seger, R.; Mullen, K.M.; van Stokkum, I.H.M. Glotaran: A Java-based graphical user interface for the R package TIMP. *J. Stat. Softw.* **2012**, *49*, 22.
4. Fang, C.; Frontiera, R.R.; Tran, R.; Mathies, R.A. Mapping GFP structure evolution during proton transfer with femtosecond Raman spectroscopy. *Nature* **2009**, *462*, 200-204.
5. Shu, X.; Leiderman, P.; Gepshtein, R.; Smith, N.R.; Kallio, K.; Huppert, D.; Remington, S.J. An alternative excited-state proton transfer pathway in green fluorescent protein variant S205V. *Protein Sci.* **2007**, *16*, 2703-2710.
6. Hoffman, D.P.; Mathies, R.A. Femtosecond stimulated Raman exposes the role of vibrational coherence in condensed-phase photoreactivity. *Acc. Chem. Res.* **2016**, *49*, 616-625.
7. Fang, C.; Tang, L.; Oscar, B.G.; Chen, C. Capturing structural snapshots during photochemical reactions with ultrafast Raman spectroscopy: From materials transformation to biosensor responses. *J. Phys. Chem. Lett.* **2018**, *9*, 3253-3263.
